# The co-application of nitrogen and phosphorus improved nutrient uptake and productivity of *Ipomoea batatas* plants grown in saline-calcareous soils

**DOI:** 10.1038/s41598-025-03375-z

**Published:** 2025-05-26

**Authors:** Abdel-Salam A. Kamel, Mohamady I. El-Kherbawy, Ahmed A. M. Awad, Atef A. A. Sweed

**Affiliations:** 1 Soil and Natural Resources Department, Faculty of Agriculture and Natural Resources, Aswan University, Aswan, 81528 Egypt; 2 Soil and Water Department, Faculty of Agriculture, Cairo University, PO Box 12613, Giza, Egypt

**Keywords:** Leaf nutrient composition, Ion homeostasis, Saline calcareous soil, Sweet potato plants, Physiological growth attributes, Yield and its components, Sustainability, Environmental sciences, Plant development, Plant physiology, Plant reproduction, Plant stress responses

## Abstract

A combination of abiotic stresses, such as salinity and calcification, can be even more destructive to crop performance than the effects of each type of stress alone. Two field experiments (in 2022 and 2023) attempted to explore the individual impact of three nitrogenous fertilizer types (NFTs, including ammonium nitrate, urea, and ammonium sulfate: N_1_, N_2_, and N_3_, respectively) and three other phosphate fertilizer types (PFTs, including calcium super-phosphate, mono-ammonium phosphate, and urea phosphate: P_1_, P_2_, and P_3_, respectively) and their interactive effects on nutritional status, physiological growth attributes, yield, and the quality attributes of sweet potato (Beauregard cv.) plants cultivated in saline–calcareous soil. This study was carried out using a split-plot system in a randomized complete block design with three replications. The results obtained for both seasons indicated that ammonium sulfate was the superior fertilizer in terms of its effects on leaf macronutrient contents, except for those of nitrogen and magnesium, which were the most impacted when ammonium nitrate and urea were used, respectively. In addition, plants fertilized with N_1_ demonstrated the highest ion homeostasis values, plant height, and calcium and magnesium contents in tuberous roots. Likewise, applying P_3_ was the most impactful on leaf macro- and micronutrient content, except for that of calcium, as well as magnesium and copper, which were the most affected when P_1_ and P_2_ were used, respectively. The use of P_3_ also produced the best results in terms of SPAD readings, leaf area, tuberous root volume, diameter, and total root yield. Furthermore, after applying P_1_, we recorded the highest ion homeostasis values, tuberous potassium, and calcium content. The coupled application of N_1_ and N_3_ with both P_2_ and P_3_ was the most effective in most of the characteristics we studied.

## Introduction

Extreme population growth is one of the major constraints for achieving economic development goals, especially in developing countries. It has been estimated that the world’s population will exceed 9.7 billion by 2064, increasing food demand by as much as 70%^1^. In this context, Egypt is one of the developing countries that suffers from food insecurity resulting from increasing population growth. Egypt’s population is expected to exceed 150 million in 2050. In addition, Egypt’s limited arable lands do not exceed 6% of the country’s total area of 1,000,000 km^2^ (approximately 1,019,600 km^2^^2,3^. In addition, large areas of arable land are vulnerable to degradation due to poor agricultural practices, inappropriate anthropogenic activities, and climate change in arid and semi-arid zones^4,5^. In arid and semi-arid regions, among the various abiotic stressors, the salinity and calcification of soil are considered major interrelated factors that can limit crop productivity^6,7^. Soil salinity is the environmental factor that most negatively affects soil fertility and plant growth, which, in turn, affects crop yield through water stress and cytotoxicity owing to the excessive absorption of sodium (Na^+^) and chloride (Cl^-^) ions^8–11^. In addition, several harmful impacts on the physiological, biochemical, and morphological characteristics of plants, such as their ability to uptake water and nutrients, their photosynthetic performance, and their antioxidant mechanisms, have been observed to occur primarily because of high levels of Na^+^ in soil^12–15^. It is estimated that approximately 33% of irrigated land and over 20% of the world’s cultivated land will be affected by salt by the middle of the 21 st century^16^. Moreover, it is predicted that about half of the world’s agricultural land will be destroyed by salt and that 1.5 million hectares of land will remain unused for agricultural production annually^17^. The calcification of soil has equally severe impacts as salinity. Calcareous soil is defined as soil containing an amount of calcium carbonate (CaCO_3_) ranging between 3 and 95%^18^. This poses a challenge to plant growth and development because of its profound effect on the formation of impermeable layers that impair water infiltration and root growth^19^. These layers occur naturally in the hemisphere (in arid and semi-arid regions) and cover more than one-third of the Earth’s land surface, especially in arid and semi-arid regions^20^. In Egypt, saline–calcareous soils cover a considerable portion of the country: roughly 27–36% of its agricultural lands or approximately 273,000 hectares. The high alkalinity (ranging from 7.6 to 8.4) of these soils may result in low solubility and the low availability of their macronutrients and micronutrients due to nutrient imbalance^21,22^. Other negative properties of calcareous soils have been highlighted in several previous studies, including a low water-holding capacity, a low cation exchange capacity, low contents of clay and organic matter, the crusting and cracking of surface layers, and the loss of nutrients^23,24^.

Therefore, several alternative fertilization approaches have been created to mitigate the harmful impacts of salinity and calcification stresses. In this vein, it has been shown that balanced nutrition with both nitrogen (N) and phosphorus (P) provides the best nutrients for plant performance. N is the most important macronutrient, followed by P. Both of them are more important than the other major nutrients due to their significant role in various critical physiological and biochemical processes that increase yield production and its components^25^. Further, a fertilizer containing both nutrients can improve nutrient uptake through the fertilizer’s crucial effect in enhancing the volume, area, diameter, and length of roots. N is an essential nutrient for several plant molecules such as amino acids, proteins, chlorophyll, nucleic acids, and phytohormones and is involved in carbon and nitrogen metabolism. Therefore, if an insufficient amount of N is available to plants, it can hinder their growth. Conversely, the adequate application of N can increase crop quality yield^26^ and promote plants’ ability to tolerate harsh environmental conditions such as limited water availability under saline conditions. Excessive use of N may cause its loss through volatilization in ammonia (NH_4_^+^) and nitrate (NO_3_^−^) forms, resulting in a harmful ecosystem^27^. Likewise, P is one of the most limiting nutrients in agricultural production systems, specifically under conditions of abiotic stress^28^. Generally, it is taken up through plant roots as a monovalent (H_2_PO_4_^−^) and divalent (HPO_4_^−−^) chemical. As previously known, most Egyptian soils are rich in inorganic P, but the vast majority of this P is strictly fixed due to its precipitation in calcium phosphate form^29^, accordingly, small proportions of inorganic P are available for plants. The fundamental role of P in various physiological and biochemical processes has been reported in numerous studies. In their study^30^ reported that it is an essential element in a wide range of metabiotic processes such as the synthesis of nucleic acids and energy-rich compounds.

Sweet potato, *Ipomoea batatas* L., is one of the most important tuberous root crops that play a paramount role in food security, animal feed, and industrial raw materials in most developing countries^31^. Globally, sweet potato crops rank as the third most crucial tuberous crop after potato and cassava^32^. To date, to the best of our knowledge, sweet potato’s tuberous roots are distinguished by their high nutritional value, including their high content of carbohydrates, lipids, and proteins. They are also a good source of carotene, vitamins, and minerals in the human diet^33,34^. Therefore, our research aimed to investigate the individual and interactive impacts of different nitrogenous and phosphate fertilizer types on the composition of leaf nutrients, ion homeostasis, physiological growth attributes, tuberous root yield, and the quality of sweet potato cultivated in a semi-arid, saline–calcareous environment.

## Materials and methods

### Study location overview, timing, plant material, and climatic conditions

Two field trials were conducted on the experimental farm of the Faculty of Agriculture and Natural Resources, Aswan University, Egypt. This farm is located between 24° 05` 20"N and 32° 53’ 59"E, and the trials were conducted during 2022 and 2023 seasons. This research was carried out to assess the potential performance of three nitrogen fertilizers and three phosphorus fertilizers on sweet potato (*Ipomoea batatas* L. Beauregard cv.) cultivated under a drip irrigation system in sandy soil characterized by undesirable properties such as high salt concentration and high CaCO_3_ content (Table 1). The tested variety was obtained by collecting vine cuttings from the AGROFOOD Farm in the An Nubariyah district, El-Behira province, Egypt. The average climatic data of both growing seasons (from April to September) of the study region are presented in Table 2.

**Table 1:** Some chemical and physical properties of the soil tested in the study in 2022 and 2023.

Soil properties	Unit	2022	2023	References
Particle size distribution				
Sand	%	91.45	91.60	^35^
Silt		3.75	3.50	
Clay		4.80	4.90	
Soil texture		Sandy	Sandy	
pH in soil paste		7.54	7.65	^36^
ECe in soil paste extract	dS m^−1^	7.74	8.90	^37^
OM	%	0.57	0.92	^38^
CaCO_3_		19.20	19.50	^37^
Soluble ions (cations and anions)				
Na^+^	meq L^−1^	63.40	69.60	^39^
K^+^		0.21	1.01	
Ca^2+^		6.50	7.00	
Mg^2+^		4.00	6.00	
CO_3_^2−^		0.00	0.00	^36^
HCO_3_^−^		4.20	3.60	
Cl^−^		54.41	60.03	
SO_4_^2−^		15.5	18.00	
Available macronutrients				
N	mg kg^−1^	160.00	201.00	^40^
P-Extractable with NaHCO_3_ pH 8.5		1542.00	1722.00	^39^
P-Extractable with NH_4_AC pH 7.0		169.00	198.00	
Available micronutrients				^41,42^
Fe-Extractable with DTPA	mg kg^−1^	38.40	39.50	
Mn- Extractable with DTPA		17.60	18.40	
Zn- Extractable with DTPA		10.60	11.40	
Cu- Extractable with DTPA		3.80	4.20	

EC-electrical conductivity. OM-organic matter.

**Table 2:** Average monthly of weather data for the Aswan Province, Egypt for during the sweet potato growing seasons (2022 and 2023).

Month	ADT	ANT	ARH	U_2_	AP
	(°C)		(%)	(ms^−1^)	(mm.d^−1^)
2022 growth season					
April	29.15	26.82	80.69	4.34	2.50
May	29.19	27.15	77.25	4.12	0.49
June	30.22	27.08	77.88	4.31	2.08
July	29.33	25.62	81.38	5.04	3.11
August	28.97	26.70	78.88	4.41	0.83
September	29.22	26.62	76.75	4.84	0.55
2023 growth season					
April	29.23	26.04	81.19	3.17	10.28
May	29.79	26.90	81.38	3.54	7.45
June	29.70	26.92	79.25	3.57	4.48
July	29.95	26.94	81.31	3.71	4.09
August	29.55	27.24	78.56	4.16	3.96
September	29.59	27.58	79.44	4.69	4.20

*ADT* and *ANT* the average of day and night temperatures, respectively. *ARH* average of relatively humid, *U*_*2*_ average of wind speed, and *AP* average precipitation.

### Treatments, design of experiments, transplanting, and intercultural operations

According to technical bulletin No. 11, issued in 2013 by a ministry in Egypt, the fertilization program of sweet potato grown in sandy soil includes the application of a fertilization ratio of 15 kg *N* + 31.0 kg P_2_O_5_ + 50 kg K_2_O obtained from ammonium nitrate NH_4_NO_3_ *N* ~ 33.0%, granular calcium super-phosphate Ca(H_2_PO_4_)_2_ P_2_O_5_ ~ 15.5%, and potassium sulfate K_2_SO_4_ K_2_O ~ 48–52% as nitrogen (N), phosphorus (P), and potassium (K), respectively. In our research, we applied the recommended fertilization ratio (15 *N* + 31.0 P_2_O_5_ + 50 K_2_O) with three nitrogenous fertilizers types (NFTs)—ammonium nitrate NH_4_NO_3_ *N* ~ 33.0% (AN), urea H_2_N-CO-NH_2_ *N* ~ 46.5% (UR), and ammonium sulfate (NH_4_)_2_SO_4_ *N* ~ 20.6% (AS)—and three phosphate fertilizer types (PFTs)—granular calcium super-phosphate (GCSP) Ca(H_2_PO_4_)_2_ P_2_O_5_ ~ 15.5%, mono-ammonium phosphate (MAP; 12–61-0), and urea-phosphate (UP; 17.5–44-0). Both types of fertilizers contain nitrogen and phosphorus. All treatments were applied as a soil application three times: 30, 45, and 60 days after transplanting (DAT) cuttings. The details of the treatments applied in this study are shown in Table 3. The results of the chemical and elemental analysis of fertilizers carried out in this research are presented in Table 4. The main experimental plots were identified according to the three nitrogenous fertilizers.

**Table 3:** The details of the experimental treatments.

Nitrogen	Phosphorus	Details of treatments	Applying time	Symbol
N_1_	P_1_	50.0 kg AN + 200.0 kg GCSP + 100 kg KS	All treatments were applied three times after 30, 45 and 60 days after transplanting (DAT)	NP_11_
	P_2_	30.0 kg AN + 51.0 kg MAP + 100 kg KS		NP_12_
	P_3_	10.3 kg AN + 71.0 kg UP + 100 kg KS		NP_13_
N_2_	P_1_	34.50 kg U + 200.0 kg GCSP + 100 kg KS		NP_21_
	P_2_	21.20 kg U + 51.0 kg MAP + 100 kg KS		NP_22_
	P_3_	7.20 kg U + 71.0 kg UP + 100 kg KS		NP_23_
N_3_	P_1_	77.67 kg AS + 200 kgGCSP + 100 kg KS		NP_31_
	P_2_	48.00 kg AS + 51.00 kg GCSP + 100 kg KS		NP_32_
	P_3_	16.10 kg AS + 71.00 kg GCSP + 100 kg KS		NP_33_

N_1_, N_2_ and N_3_ indicate the ammonium nitrate (AN), urea (U), and ammonium sulfate (AS), respectively. P_1_, P_2_ and P_3_ indicate the granular calcium super phosphate (GCSP), mono-ammonium phosphate (MAP), and urea-phosphate (UP), respectively. KS indicate potassium sulfate (K_2_SO_4_).

**Table 4:** Chemical and elemental analysis of fertilizers applied in the study.

Properties	*N*_1_	*N*_2_	*N*_3_	*P*_1_	*P*_2_	*P*_3_
Chemical formula	NH_4_NO_3_	H_2_N-CO-NH_2_	(NH_4_)_2_SO_4_	Ca(H_2_PO_4_)_2_	NH_4_H_2_PO_4_	CO(NH_4_)_2_.H_3_PO_4_
pH (1% solution)	5.09	10.67	4.96	7.5	4.5	1.8
N (%)	33.50	46.50	20.60	0.00	12.0	17.75
P (%)	0.00	0.00	0.00	15.50	61.0	44.0

N_1_, N_2_ and N_3_ indicate the ammonium nitrate (A-N), urea (U), and ammonium sulfate (A-S), respectively. P_1_, P_2_ and P_3_ indicate the granular calcium super phosphate (GCSP), mono-ammonium phosphate (MAP), and urea phosphate (UP), respectively. KS potassium sulfate (K_2_SO_4_).

The size of each main plot was 10.5 m x 6.0 m (63.0 m^2^. Each main plot was subdivided into three subplots of three phosphorus fertilizers. The size of each subplot was 3.5 m x 2 m (7.0 m^2^. There were 3 × 3 = 9 treatments. There were four rows in each subplot, and each row contained 10 vine cuttings planted 30 cm apart, with a total 40 plants per subplot and 120 plants in each main plot. In both growing seasons, the experiments were carried out according to the split-plot system for a randomized complete block design (RCBD), with three replicates.

### Soil sampling and determinations

Before the application of treatments, surface soil samples were collected from the surface layer at 0–30 cm and transported to the Soil, Water and Plant Laboratory at the Faculty of Agriculture and Natural Resources, Aswan University, to determine chemical and physical properties of the tested soil; these are presented in Table 1.

### Physiological and growth characteristics

A total of 10 plants were randomly collected from each experimental unit (subplot) after 90 DAT and marked for every replicate to determine characteristics. Relative chlorophyll content (SPAD reading) was measured using an SPAD 502 m device (Minolta, Osaka, Japan). The average height of these 10 plants was measured using a gradual rule (in centimeter). Both the fresh weight (FrW-P) and dry matter of leaves (DrM-P) were measured (g plant^−1^) using a digital electronic balance, and then, the leaf dry matter percentage (%LDrM) was calculated according to the following equation:

$$\% {\text{DrM}} - {\text{L }}=~\frac{{\left( {{\text{FrW}} - {\text{L}}} \right) - \left( {{\text{DrM}} - {\text{L}}} \right)}}{{\left( {{\text{DrM}} - {\text{L}}} \right)}} \times {\text{ 1}}00.$$

The leaf area (LA-P) was determined using a planimeter device.

### Evaluation of leaves and tuberous root macro/micronutrient

At 90 days after transplanting (DAT), both the fourth and fifth leaves were collected, and at 125 DAT, a random sample of roots was collected from five plants from each treatment, washed with distilled water, cleaned, sliced, and oven dried at 70 ºC for 48 h. The sample was crushed to determine macro-and micronutrients. This procedure was carried out according to the method described by^43^. The micronutrients, such as Fe, Mn, Zn, and Cu, were measured using inductively coupled plasma optical emission spectrometry (ICP-OES, Perkin-Elmer OPTIMA-2100 DV, Norwalk, CT, USA) in the Faculty of Agriculture and Natural Resources, Aswan University.

### Tubers’ chemical and physical measurements

At 125 days following harvest, the tubers were randomly picked from all remain plants in each experimental unit, cleaned, washed, weighed using a digital electronic balance to calculate the tuberous root fresh weight (FrW-T), and oven dried at 70º C. The tubers were weighed using the same digital electronic balance to calculate the tuber root dry matter (DrM-T). The tuber root dry matter percentage was calculated using the following equation: %DrM-T=$({\text{FrW}} - {\text{T}}) - ({\text{DrM}} - {\text{T}})/({\text{DrM}} - {\text{T}})$. Using a precision graduated rule, the average tuber root diameter (TRD, cm) was determined, while the average tuberous root volume (TRV, cm^3^ was measured with the help of a graduated glass cylinder and water. The total tuber root yield (TRY, t ha^−1^) was calculated using all the plants in all the subplots.

### Statistical analysis

The analysis of variance (ANOVA) in both seasons was conducted according to the split-plot structure and in a randomized, completed block design that was calculated using the Infostat statistical package, version 9.2 (INFOSTAT, 2019) according to Di Rienzo et al. (2011)^44^. The differences among all treatments were compared at *p* ≤ 0.05 using the Duncan multiple range. The standard error (SE) was calculated (Means ± SE) using Microsoft Excel 2016. Pearson’s correlation and stepwise regression were calculated using the IBM SPSS statistics 2021 Wizard.

## Results

### Leaves’ nutrients content

#### The individual impact of NFTs

**Fig. 1:**
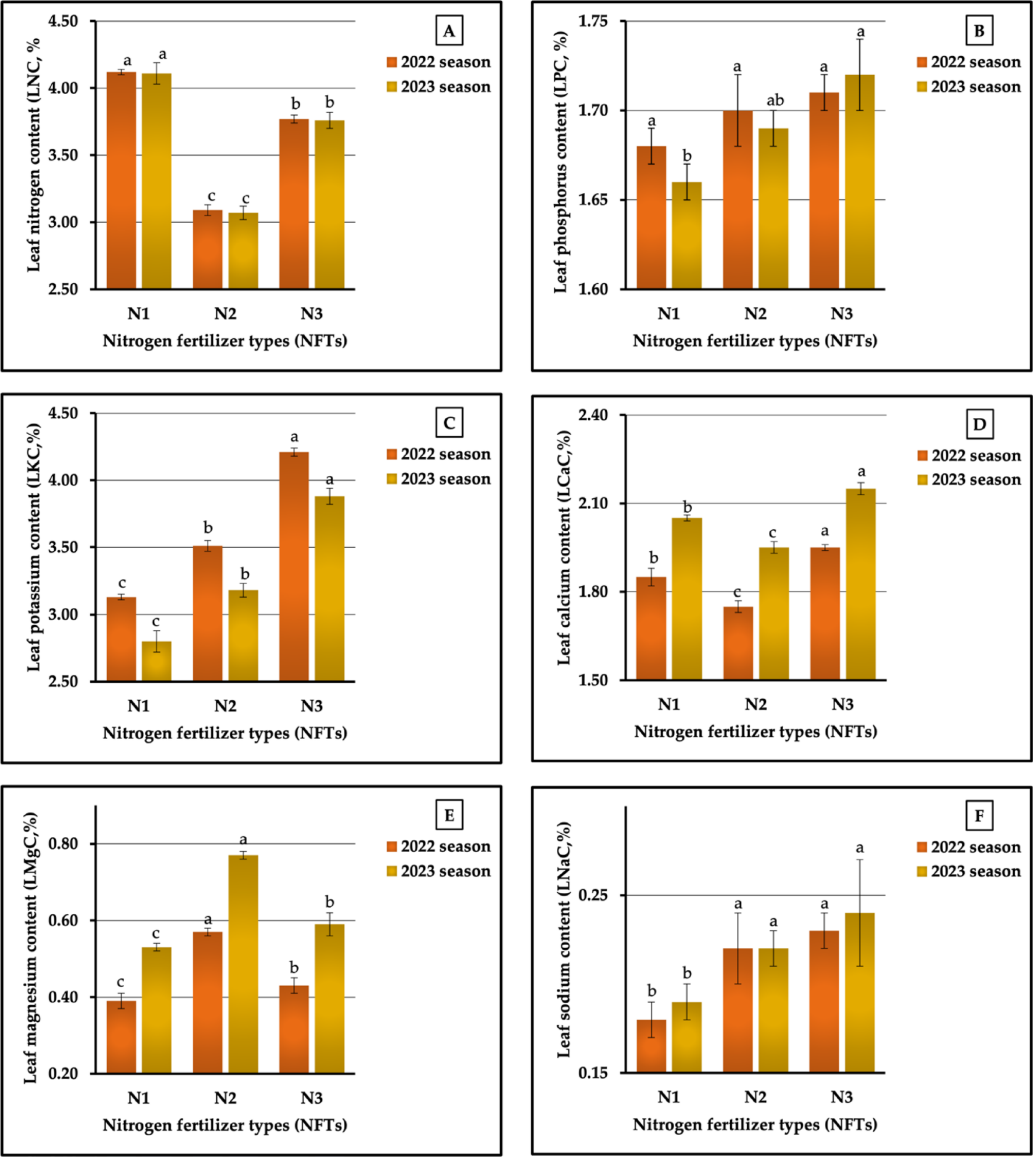
(A-F). The individual impact of nitrogen fertilizer types (NFTs) on leaf macronutrients content; **(A)** LNC, **(B)** LPC, **(C)** LKC, **(D)** LCaC, **(E)** LMgC and **(F)** LNaC on sweet potato plants cultivated in saline-calcareous soil during 2022 and 2023 growing seasons. Bars sharing the same letter in each columns denote not significantly different among treatments according to the Duncan’s Multiple range test at *p*≤0.05.

The data pertaining to the effect of the studied N-fertilizers on leaf macronutrients content in 2022 and 2023 seasons, respectively, are graphically illustrated in Fig. 1 (A-F). The obtained results revealed that the leaf nitrogen content values (LNC; 4.12 vs. 4.11%) were obtained in plants treated with ammonium nitrate (N_1_) in 2022 and 2023 seasons, respectively. Meanwhile, the highest values in the leaf content of phosphorus (LPC), potassium (LKC), calcium (LCaC), magnesium (LMgC), and sodium (LNaC) were produced using ammonium sulfate (N_3_) and recorded as 1.71 vs. 1.72% for LPC, 4.21 vs. 3.88% for LKC, 1.95 vs. 2.15% for LCaC, 0.57 vs. 0.77% for LMgC, and 0.23 vs. 0.22% for LNaC in both growing seasons, respectively. On the contrary, the application of urea (N_2_) produced the lowest LNC and LCaC: these were recorded as 3.09 vs. 3.07% and 1.75 vs. 1.95% in 2022 and 2023 seasons, respectively. The application of N_1_ was the least influential on LPC, LKC, LMgC, and LNaC, producing 1.68 vs. 1.66%, 3.13 vs. 2.80%, 0.39 vs. 0.53%, and 0.18 vs. 0.17% in the first and second seasons, respectively.

According to the highest and lowest values, the increments in percentage were 33.33 vs. 33.88 for LNC, 1.79 vs. 3.62% for LPC, 34.50 vs. 33.57% for LKC, 11.43 vs. 10.26% for LCaC, 46.15 vs. 45.28% for LMgC, and 27.78 vs. 29.41% in the two growing seasons, respectively. The results of the ANOVA showed that all N fertilizer treatments had a highly significant effect on all leaf macronutrient contents except on those of LPC, in which case the effects were non-significant and significant in 2022 and 2023 seasons, respectively.

**Fig. 2:**
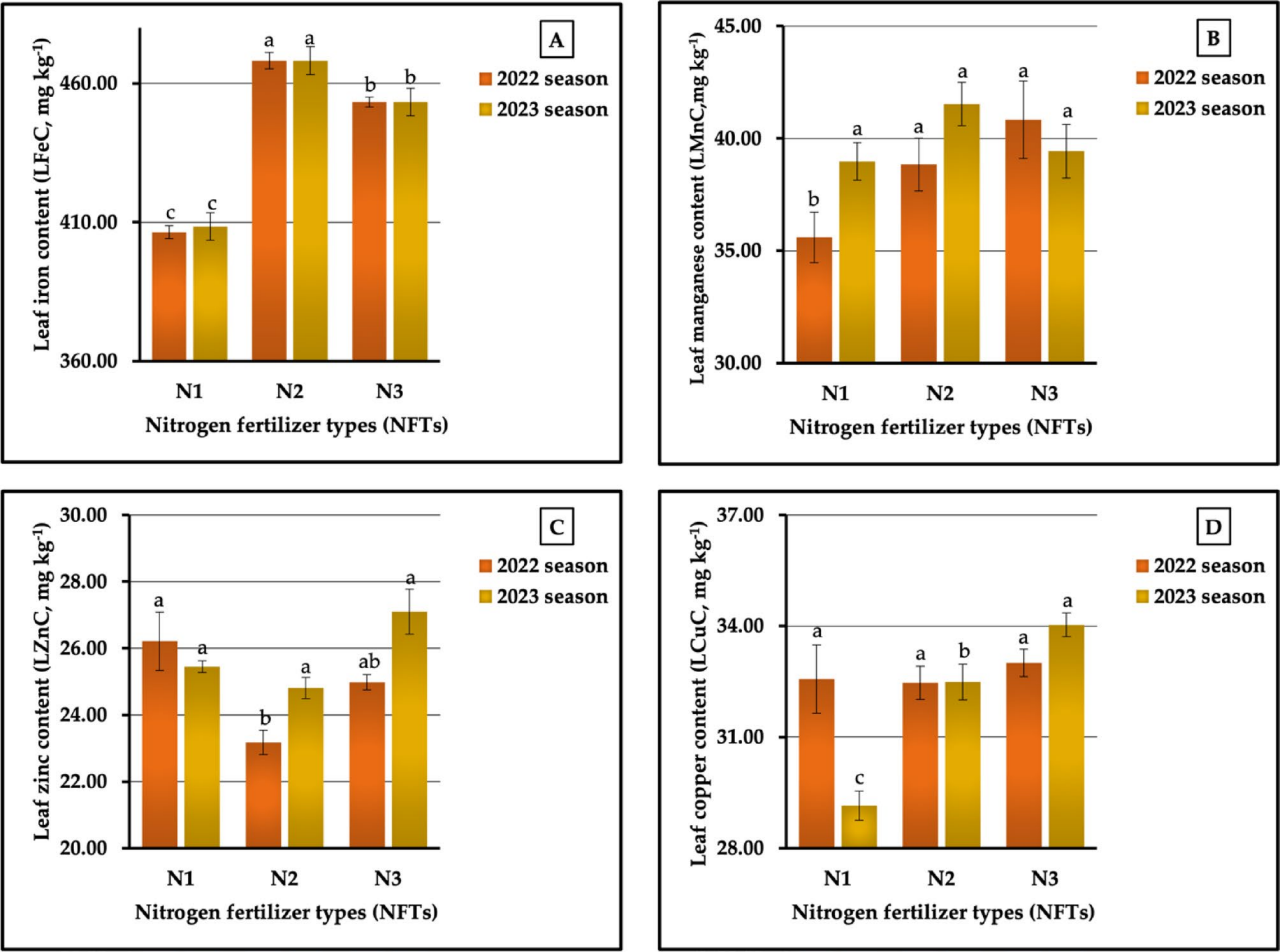
(A-D). The individual impact of nitrogen fertilizer types (NFTs) on leaf micronutrients content; **(A)** LFeC, **(B)** LMnC, **(C)** LZnC, and **(D)** LCuC on sweet potato plants cultivated in saline-calcareous soil during 2022 and 2023 growing seasons. Bars sharing the same letter in each columns denote not significantly different among treatments according to the Duncan’s Multiple range test at *p*≤0.05.

The results obtained from our field research, graphically represented in Fig. 2 (A-D), showed that N₂ was the superior treatment for leaf iron content ((LFeC) 468.17 vs. 470.22 mg kg^−1^) in 2022 and 2023 seasons, respectively, and for leaf manganese content (LMnC), recorded at 41.53 mg kg^−1^ in the second season. However, the same treatment had the least influence on the leaf zinc content (LZnC) in both seasons (recorded as 23.18 vs. 24.81 mg kg^−1^) and on the leaf copper content (LCuC) in the first season (recorded as 32.47 mg kg^−1^). It is evident from Fig. 2 (A-D) that the sweet potato plants fertilized with N_3_ produced the highest LMnC (40.83 mg kg^−1^) in the first season, the highest LZnC (27.10 mg kg^−1^) in the 2023 season, and the highest LCuC (33.01 vs. 34.03 mg kg^−1^) in both seasons, respectively. Meanwhile, the lowest LFeC (356.42 vs. 358.47 mg kg^−1^) and LMnC (35.60 vs. 38.97 mg kg^−1^) in both growing seasons, respectively, were obtained in plants treated with N_1_. As observed in Fig. 2 (A-D), the percentages of increase compared with the lowest values were 31.35 vs. 31.17% for LFeC, 14.69 vs. 6.57% for LMnC, 13.07 vs. 9.23% for LZnC, and 1.66 vs. 16.74% for LCuC in the two growing seasons, respectively. There were significant differences (at *p* ≤ 0.01) for LFeC in both seasons and for LCuC in the second season, while there were significant differences (at *p* ≤ 0.05) among the treatments on LMnC and LZnC in the first season and no significant effects on LCuC in 2022 season or on LMnC and Lane in 2023 season.

#### The individual impact of PFTs

**Fig. 3:**
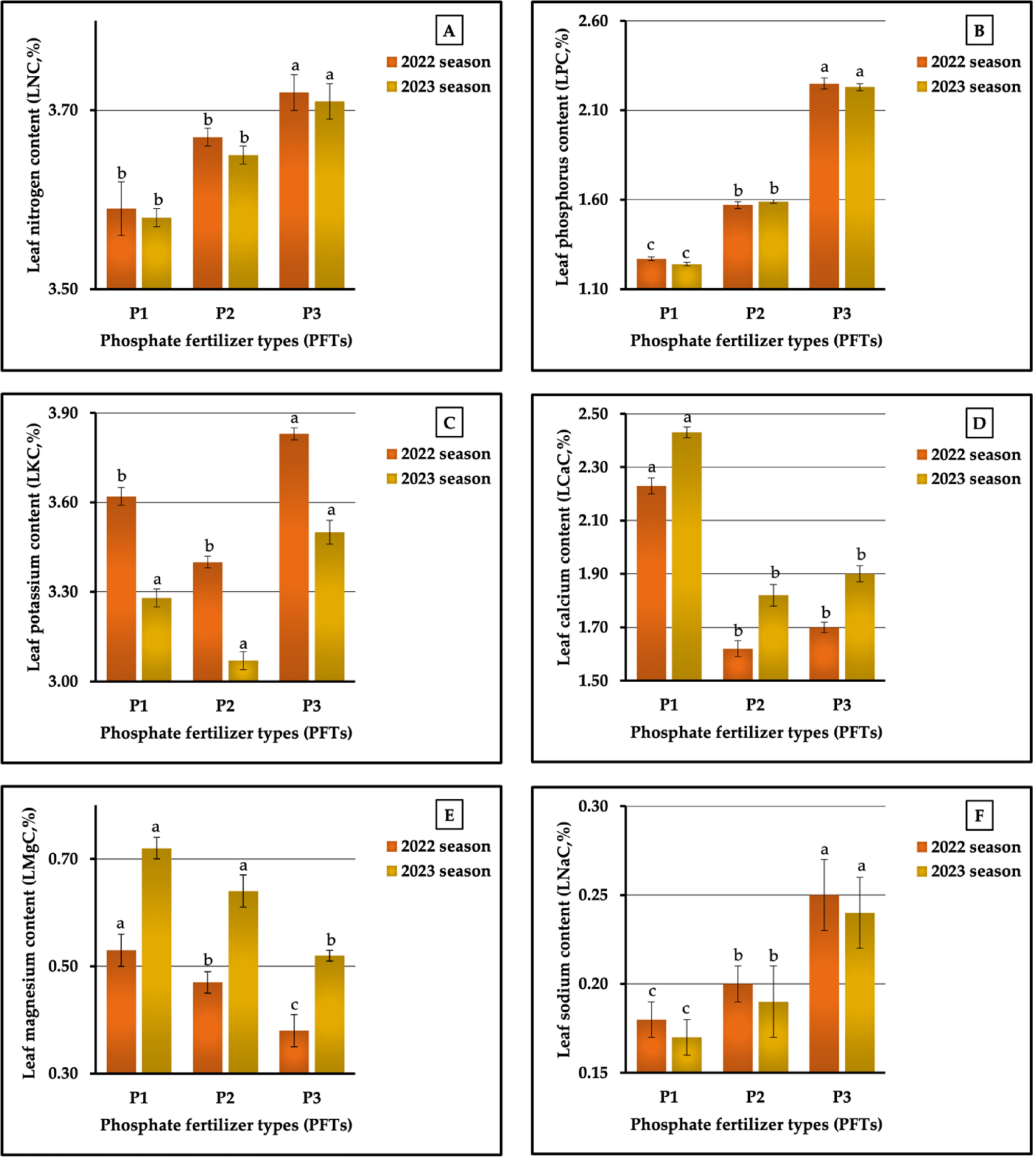
(A-F). The individual impact of phosphate fertilizer types (PFTs) on leaf macronutrients content; **(A)** LNC, **(B)** LPC, **(C)** LKC, **(D)** LCaC, **(E)** LMgC) and **(F)** LNaC on sweet potato plants cultivated in saline-calcareous soil during 2022 and 2023 growing seasons. Bars sharing the same letter in each columns denote not significantly different among treatments according to the Duncan’s Multiple range test at *p*≤0.05.

The findings of our field experiments, as seen in Fig. 3 (A-F) reported that the highest LNC, LPC, LKC, and LNaC values were obtained applying urea-phosphate (P_3_), which produced values of 3.72 vs. 3.71%, 2.25 vs. 2.23, 3.83 vs. 3.50%, and 0.27 vs. 0.26% in both seasons, respectively, while the application of granular calcium super-phosphate (P_1_) was the superior phosphorus fertilizer for LCaC and LMgC, producing 2.23 vs. 2.43% and 0.53 vs. 0.72 in 2022 and 2023 seasons, respectively. However, fertilization of plants with P_1_, was the least impactful, resulting in the lowest values of LNC (3.59 vs. 3,58%), LPC (1.27 vs. 1.24%), and LNaC (0.18 vs. 0.17%) in both seasons, respectively. Similar results were observed regarding the lowest values. However, the plants fertilized with mono-ammonium phosphate (P_2_) produced the lowest LKC (3.40 vs. 3.07%) and LCaC (1.62 vs. 1.82%), while the lowest values in LMgC (0.38 vs. 0.52% in the first and second seasons, respectively) were recorded in plants fertilized with P_3_. In terms of a comparison between the highest and lowest values, the rates of increase were 3.62 vs. 3.63% for LNC, 77.17 vs. 79.84% for LPC, 12.65 vs. 14.01% for LKC, 37.65 vs. 33.52% for LCaC, 39.47 vs. 38.46% for LMgC, and 50.00 vs. 52.94% for LNaC in the two growing seasons, respectively. The results obtained from the statistical analysis showed significant impacts (at *p* ≤ 0.01) on LPC, LCaC, and LMgC in both seasons and on LNC in the second season. In addition, significant. impacts (at *p* ≤ 0.05) on LNC and LKC were observed in the first season.

**Fig. 4:**
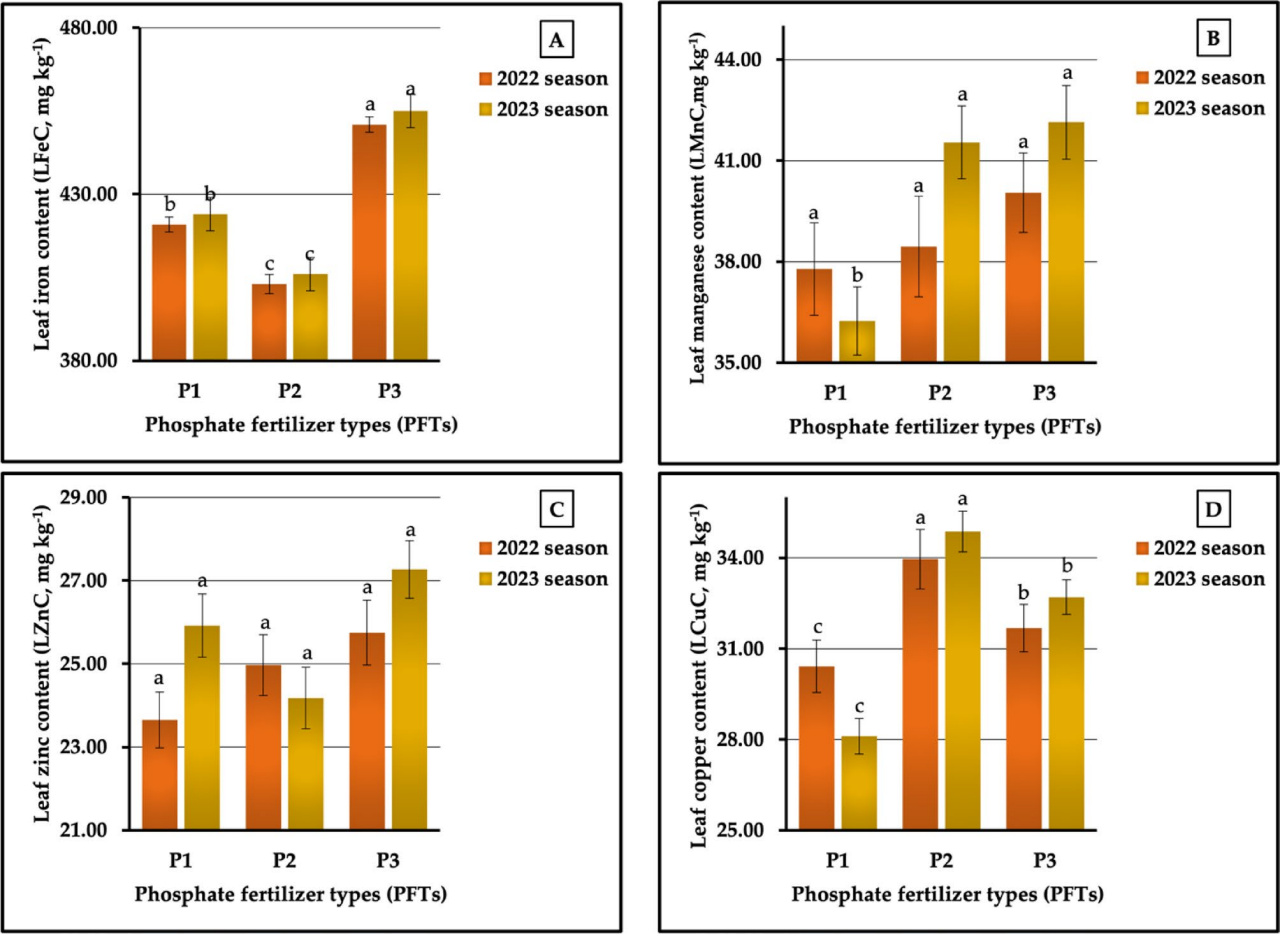
(A-D). The individual impact of phosphate fertilizer types (PFTs) on leaf micronutrients content; **(A)** iron (LFeC), **(B)** manganese (LMnC), **(C)** zinc (LZnC), and **(D)** copper (LCuC) on sweet potato plants cultivated in saline-calcareous soil during 2022 and 2023 growing seasons. Bars sharing the same letter in each columns denote not significantly different among treatments according to the Duncan’s Multiple range test at *p*≤0.05.

The data in Fig. 4 (A-D) indicate the impact of different phosphorus fertilizers on leaf micronutrient content. The general trend of our results revealed that the plants treated with P_3_ demonstrated the highest LFeC, LMnC, and LZnC levels, producing 450.92 vs. 454.97 mg kg^−1^, 40.05 vs. 42.14 mg kg^−1^, and 25.75 vs. 27.27 mg kg^−1^ in both growing seasons, respectively.

Furthermore, the highest LCuC (33.96 vs. 34,87 mg kg^−1^) content values were obtained in plants fertilized with P_1_. Similar results were observed for the lowest values of Fe, Mn, Zn, and Cu content, since the lowest LFeC (403.61 vs. 406.06 mg kg^−1^) in both seasons and the lowest LZnC (24.18 mg kg^−1^) in 2023 growing season were produced in plants that were treated with P_2_. Similarly, plants that were treated with P_1_ demonstrated the lowest values of LMnC (37.78 vs. 36.24 mg kg^−1^) and LCuC (30.42 vs. 28.11 mg kg^−1^) in 2022 and 2023 seasons, and plants that were treated with P_1_ demonstrated the lowest LZnC (23.65 mg kg^−1^) level in the first season. The results obtained from the statistical analysis indicated highly significant differences for LFeC and LCuC and no significant impacts on LZnC in both seasons. There were non-significant and significant effects on LMnC in the two growing seasons, respectively. The increment rates of the highest and lowest values were 11.89 vs. 12.05%, 6.01 vs. 16.28%, 8.88 vs. 12.78%, and 11.64 vs. 24.05% for LFeC, LMnC, LZnC, and LCuC in both seasons, respectively.

#### The impact of the NFT X PFT interaction

The results related to the effect of interaction between nitrogenous and phosphorus fertilizers on leaf macronutrient content in 2022 and 2023 growing seasons are presented in Table 5. The results we obtained revealed that sweet potato plants treated with ammonium nitrate and urea-phosphate (N_1_P_3_) demonstrated the highest values (4.43 vs. 4.42%) of LNC in 2022 and 2023 seasons, respectively.

**Table 5:** Impact of interaction between nitrogenous and phosphorus fertilizer sources on leaf macronutrients content of sweet potato plants cultivated in saline-calcareous soil during 2022 and 2023 growing seasons.

Treatment		Leaf macronutrients content					
NFTs	PFTs	LNC	LPC	LKC	LCaC	LMgC	LNaC
		(%)					
2022 growth season							
N_1_	P_1_	4.04±0.06^b^	1.27±0.06^c^	3.94±0.14^b^	1.79±0.03^cd^	0.48±0.02^cd^	0.09±0.01^i^
	P_2_	3.88±0.06^b^	1.54± 0.04^b^	2.11±0.09^e^	1.83±0.04^c^	0.12±0.02^g^	0.23±0.01^d^
	P_3_	4.43±0.05^a^	2.24±0.07^a^	3.35±0.18^cd^	1.91±0.08^bc^	0.56±0.02^bc^	0.22±0.02^e^
N_2_	P_1_	3.09±0.06^d^	1.27±0.07^c^	3.19±0.12^d^	2.31±0.09^ab^	0.61±0.03^b^	0.21±0.01^f^
	P_2_	3.08±0.07^d^	1.57±0.03^b^	3.99±0.13^b^	1.67±0.09^cd^	0.87±0.02^a^	0.19±0.01^g^
	P_3_	3.08±0.07^d^	2.24±0.03^a^	3.35±0.12^cd^	1.59±0.05^cd^	0.23±0.02^f^	0.30±0.01^a^
N_3_	P_1_	3.64±0.04^c^	1.26±0.06^c^	3.72±0.13^bc^	2.59±0.04^a^	0.50±0.03^cd^	0.25±0.01^c^
	P_2_	4.04±0.06^b^	1.60±0.03^b^	4.10±0.13^b^	1.35±0.04^d^	0.44±0.02^de^	0.17±0.01^h^
	P_3_	3.64±0.04^c^	2.28±0.03^a^	4.80±0.16^a^	1.59±0.08^cd^	0.35±0.02^e^	0.28±0.01^b^
N * P		**	*	**	**	**	**
2023 growth season							
N_1_	P_1_	4.03±0.03^b^	1.27±0.03^c^	3.61±0.25^ab^	1.99±0.12^cd^	0.65±0.04^c^	0.08±0.01^i^
	P_2_	3.87±0.03^c^	1.55±0.03^b^	2.85±0.14^b^	2.03±0.09^c^	0.17±0.02^e^	0.22±0.01^d^
	P_3_	4.42±0.03^a^	2.22±0.04^a^	3.01±0.14^b^	2.11±0.06^bc^	0.76±0.05^bc^	0.21±0.01^e^
N_2_	P_1_	3.08±0.05^e^	1.26±0.05^c^	1.78±0.10^c^	2.51±0.08^ab^	0.83±0.06^b^	0.20±0.01^f^
	P_2_	3.06±0.05^e^	1.59±0.01^b^	3.66±0.23^ab^	1.55±0.08^d^	1.17±0.08^a^	0.18±0.01^g^
	P_3_	3.06±0.05^e^	2.26±0.03^a^	3.01±0.10^b^	1.79±0.09^cd^	0.32±0.02^e^	0.29±0.01^a^
N_3_	P_1_	3.63±0.03^d^	1.29±0.05^c^	3.39±0.21^ab^	2.79±0.13^a^	0.69±0.05^bc^	0.24±0.01^c^
	P_2_	4.03±0.03^b^	1.62±0.01^b^	3.77±0.21^ab^	1.87±0.09^cd^	0.59±0.04^cd^	0.16±0.01^h^
	P_3_	3.63±0.03^d^	2.26±0.04^a^	4.47±0.28^a^	1.79±0.09^cd^	0.48±0.03^d^	0.27±0.01^b^
N * P		**	*	**	**	**	**

Mean values (±SE), same lower-case letters in each columns denote not significant among treatments according to the Duncan’s Multiple range test at *p*≤0.05. NFS nitrogenous fertilizer source; N_1_, N_2_ and N_3_ represent ammonium nitrate, urea and ammonium sulfate, respectively. PFS phosphorus fertilizer source; P_1_, P_2_ and P_3_ represent granular calcium super phosphate, mono-ammonium-phosphate and urea-phosphate, respectively. LNC, LPC, LKC, LCaC, LMgC, LNaC indicate the leaf nitrogen, phosphorus, potassium, calcium, magnesium and sodium content, respectively.

Meanwhile, the application of ammonium sulfate with urea-phosphate (N_3_P_3_), ammonium sulfate with granular calcium super-phosphate (N_3_P_1_), and urea with mono-ammonium phosphate (N_2_P_2_) were the superior treatments; plants that received these treatments demonstrated the highest values in LKC (4.80 vs. 4.47%), LCaC (2.59 vs. 2.79%), and LMgC (0.87 vs. 1.17%) in both seasons, respectively. In addition, the plants fertilized with N_3_P_3_ and with urea and urea-phosphate (N_2_P_3_) displayed the highest LPC in the first and second seasons, respectively. The highest LNaC values were obtained in plants treated with N_2_P_3_ in both seasons. On the other hand, we observed that the lowest values for LKC (2.11 vs. 1.78%) and LMgC (0.12 vs. 0.17%) were recorded in plants nourished by ammonium nitrate with mono-ammonium-phosphate (N_1_P_2_). Moreover, the N₂P₂ treatment was the least impactful, producing the lowest value in LCaC (1.35 vs. 1.55%) in both seasons, respectively, and the lowest value in LNC (3.06%) in the second season. Furthermore, the lowest LPC (1.26 vs. 1.23%) values were recorded in plants treated with N_3_P_1_ and N_2_P_1_ treatments in 2022 and 2023 growing seasons, respectively. Statistically, the treatments had a significant influence (at *p* ≤ 0.05) on LPC and significant effects (at *p* ≤ 0.01) on other leaf macronutrients’ content in both seasons.

Concerning the effect of the NFT x PFT interaction on the leaf content of Fe, Mn, Zn, and Cu, the results listed in Table 6 show that the highest values of LFeC (545.51 vs. 548.56 mg kg^−1^) and LCuC (39.26 vs. 40.28 mg kg^−1^) were obtained in plants fertilized with urea and granular calcium super-phosphate (N_2_P_1_) in both growing seasons, respectively. Similarly, the combined application of urea and mono-ammonium-phosphate (N₂P_2_) was the most influential on LCuC, producing the highest values (39.26 vs. 40.28 mg kg^−1^) in 2022 and 2023 growth seasons, respectively. Dissimilar results were observed regarding the leaf content of Mn and Zn. However, the greatest LMnC (45.83 vs. 46.61 mg kg^−1^) values were achieved in plants treated with N_3_P_3_ and N_1_P₂ in both seasons, respectively. Applying N_1_P_3_ and N₂P_3_ produced the highest LZnC (30.25 vs. 28.09 mg kg^−1^) in both seasons. On the contrary, the lowest LFeC (390.51 vs. 395, 55 mg kg^−1^) and LMnC levels (32.67 vs. 30.31 mg kg^−1^) in both seasons and the lowest LCuC level (24.89 mg kg^−1^) in the second season were recorded in plants treated with ammonium nitrate with calcium superphosphate (N_1_P_1_). The increasing rates amounted to 39.69 vs. 38.68% for LFeC, 42.91 vs. 53.78% for LMnC, 41.55 vs. 28.91% for LZnC, and 45.68 vs. 61.83% for LCuC. The results of the ANOVA indicated that all treatments had highly significant impacts; in addition, highly significant impacts were observed for LMnC in the second season and LZnC in the first season.

**Table 6:** Interactive impact of NFTs and PFTs on leaf macronutrients content of sweet potato plants cultivated in saline-calcareous soil during 2022 and 2023 growing seasons.

Treatment		Leaf micronutrients content			
NFTs	PFTs	LFeC	LMnC	LZnC	LCuC
		(mg kg^−1^)			
2022 growth season					
N_1_	P_1_	291.51±0.02^g^	32.07±0.13^a^	26.43±0.04^ab^	34.86±0.04^c^
	P_2_	353.01±0.02^f^	38.68±0.14^a^	21.96±0.01^b^	30.27±0.04^e^
	P_3_	421.76±0.01^de^	39.05±0.14^a^	30.25±0.02^a^	32.57±0.03^d^
N_2_	P_1_	545.51±0.03^a^	36.15±0.14^a^	23.15±0.05^b^	30.20±0.04^e^
	P_2_	438.51±0.02^c^	42.10±0.14^a^	23.33±0.03^b^	40.26±0.06^a^
	P_3_	418.51±0.02^e^	41.26±0.15^a^	23.06±0.02^b^	26.95±0.04^f^
N_3_	P_1_	425.76±0.01^d^	45.11±0.13^a^	21.37±0.03^b^	29.19±0.04^e^
	P_2_	418.51±0.01^de^	34.56±0.13^a^	29.63±0.03^a^	34.34±0.05^c^
	P_3_	517.51±0.01^b^	45.83±0.14^a^	23.93±0.04^b^	38.51±0.03^b^
N * P		**	ns	**	**
2023 growth season					
N_1_	P_1_	295.55±0.02^i^	30.31±0.09^c^	27.81±0.02^a^	24.89±0.03^h^
	P_2_	355.06±0.02^h^	46.61±0.12^a^	21.79±0.03^a^	29.96±0.04^e^
	P_3_	424.81±0.01^e^	40.00±0.12^ab^	26.76±0.02^a^	32.59±0.06^d^
N_2_	P_1_	548.56±0.03^a^	41.94±0.10^ab^	22.74±0.02^a^	30.22±0.03^e^
	P_2_	441.56±0.02^c^	42.32±0.10^ab^	23.62±0.02^a^	40.28±0.07^a^
	P_3_	420.55±0.02^g^	40.32±0.11^ab^	28.09±0.05^a^	26.97±0.07^g^
N_3_	P_1_	427.81±0.01^d^	36.48±0.12^bc^	27.22±0.04^a^	32.59±0.04^d^
	P_2_	421.56±0.01^f^	35.69±0.11^bc^	27.13±0.03^a^	34.36±0.04^c^
	P_3_	519.56±0.01^b^	46.11±0.13^a^	26.97±0.03^a^	38.53±0.05^b^
N * P		**	**	ns	**

Mean values (±SE), same lower-case letters in each columns denote not significant among treatments according to the Duncan’s Multiple range test at *p*≤0.05. NFS nitrogenous fertilizer source; N_1_, N_2_ and N_3_ represent ammonium nitrate, urea and ammonium sulfate, respectively. PFS phosphorus fertilizer source; P_1_, P_2_ and P_3_ represent granular calcium super phosphate, mono-ammonium-phosphate and urea-phosphate, respectively. LNC, LPC, LKC, LCaC, LMgC, LNaC indicate the leaf nitrogen, phosphorus, potassium, calcium, magnesium and sodium content, respectively.

### Ion homeostasis

#### The individual impacts of NFTs

**Fig. 5:**
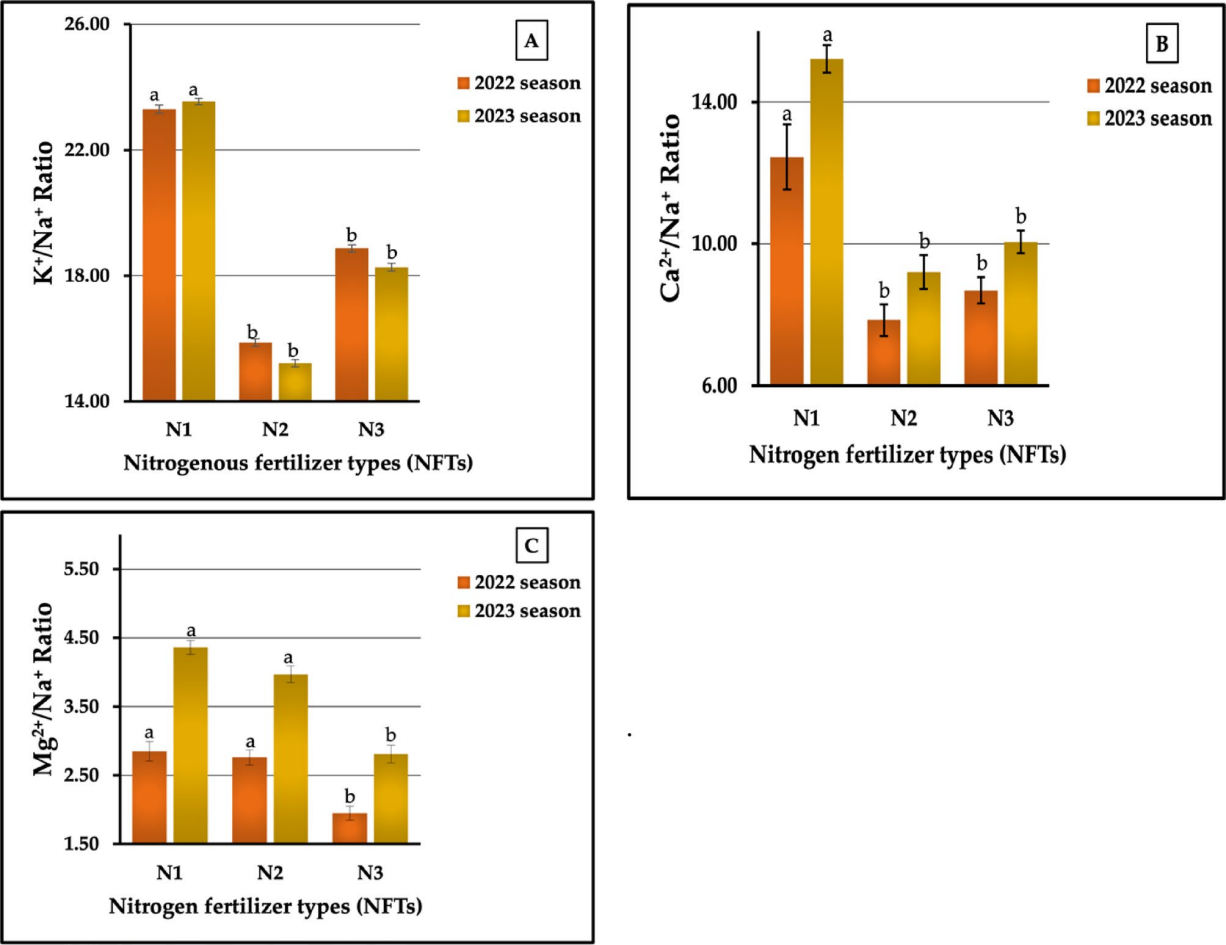
(A-C). The individual impact of nitrogen fertilizer types (NFTs) on ions homeostasis **(A)** K^+^/Na^+^, **(B)** Ca^2+^/Na^+^, and **(C)** Mg^2+^/Na^+^ ratios in leaves of sweet-potato plants cultivated in saline-calcareous soil during 2022 and 2023 growing seasons. Bars sharing the same letter in each columns denote not significantly different among treatments according to the Duncan’s Multiple range test at *p*≤0.05.

The individual effect of nitrogen fertilizer types (NFTs) on ionic homeostasis, e.g., in K^+^/Na^+^, Ca^2+^/Na^+^, and Mg^2+^/Na^+^ ratios, is graphically represented in Fig. 5 (A-C). The resulted obtained indicated that the nitrogen fertilizer types were ranked in descending order as N_1_ < N_3_ < N₂ (23.30 < 18.87 > 15.87 and 23.54 > 18.27 > 15.21 for the K^+^/Na^+^ ratio and as 12.45 > 8.69 > 7.85 and 15.21 > 10.05 > 9.21 for the Ca^2+^/Na^+^ ratio in the two growing seasons, respectively. Regarding the Mg^2+^/Na^+^ ratio, the studied NFTs can be arranged in the following order: N_1_ > N_2_ > N_3_ (2.85 > 2.76 > 1.95 and 4.36 > 3.97 > 2.81 in 2022 and 2023 seasons, respectively. Based on the highest and lowest values, the rates of increase amounted to 46.82. vs. 54.77% for the K^+^/Na^+^ ratio, 58.60 vs. 65.15% for the Ca^2+^/Na^+^ ratio, and 46.15 vs. 55.16% for the Mg^2+^/Na^+^ ratio in both seasons, respectively. The analysis of variance revealed that all treatments had significant impacts (at *p* ≤ 0.01) on the Ca^2+^/Na^+^ ratio in both seasons and on the Mg^2+^/Na^+^ ratio in the first season, while the same treatment had significant influences (at *p* ≤ 0.05) on the K^+^/Na^+^ ratio in both seasons and on the Mg^2+^/Na^+^ ratio in the second season.

#### The individual impacts of PFTs

**Fig. 6:**
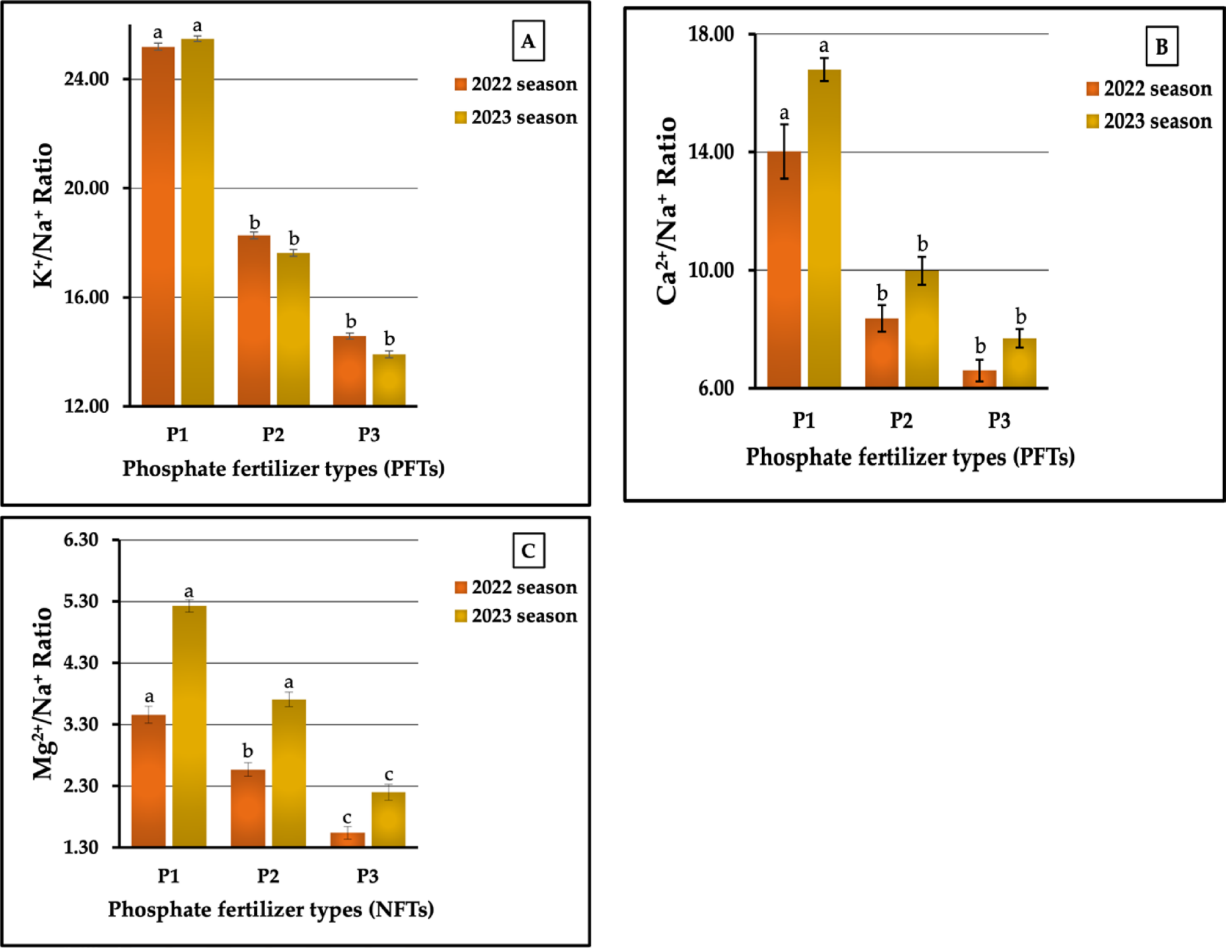
(A-C). The individual impact of phosphate fertilizer types (PFTs) on ions homeostasis **(A)** K^+^/Na^+^, **(B)** Ca^2+^/Na^+^, and **(C)** Mg^2+^/Na^+^ ratios in leaves of sweet-potato plants cultivated in saline-calcareous soil during 2022 and 2023 growing seasons. Bars sharing the same letter in each columns denote not significantly different among treatments according to the Duncan’s Multiple range test at *p*≤0.05.

The results related to the effect of PFTs on ion homeostasis are presented in Figs. 6 (A-C). Similar data were obtained for the three ion homeostasis measurements mentioned above. However, the phosphate fertilizer types, in descending order, were ranked as follows: P_1_ > P₂ > P_3_, recorded as 25.19 > 18.27 > 14.58 and 25.48 > 17.63 > 13.91 for the K^+^/Na^+^ ratio, 14.02 > 8.37 > 6.60 and 16.80 > 9.98 > 7.69 for the Ca^2+^/Na^+^ ratio, and 3.46 > 2.57 > 1.54 and 5.23 > 3.71 > 2. 20 for the Mg^2+^/Na^+^ ratio in the first and second seasons, respectively. The results presented in Fig. 6 (A-C) indicate that the increase percentages compared with the lowest values were 72.77 vs. 83.18%, 112.42 vs. 118.47, and 124.68 vs. 137.33% for the K^+^/Na^+^, Ca^2+^/Na^+^, and Mg^2+^/Na^+^ ratios in both growth seasons, respectively. The PFTs had highly significant impacts on all studied ions in both seasons.

#### The impact of NFTs X PFTs interaction

The results presented in Table 7 show that all studied ion homeostasis levels were enhanced as a result of the interaction between the nitrogen and phosphate fertilizer types. The results obtained unanimously demonstrated that the N_1_P_1_ treatment produced the highest values for all studied parameters, as we recorded levels of 45.38 vs. 47.82 for the K^+^/Na^+^ ratio, 20.59 vs. 26.15 for the Ca^2+^/Na^+^ ratio, and 5.47 vs. 8.61 for the Mg^2+^/Na^+^ ratio in both growth seasons, respectively. On the other hand, the lowest values in the K^+^/Na^+^ and Mg^2+^/Na^+^ ratios were observed in plants fertilized with N_1_P_2_: 11.08 vs. 10.24 and 0.52 vs. 0.80. N_2_P_3_ treatment was the least impactful on the Ca^2+^/Na^+^ ratio, producing 5.33 vs. 6.22 in both seasons, respectively. The analysis of variance indicated that all interactive treatments had highly significant effects on the three studied ion homeostasis levels in the two growing seasons. The results listed in Table 7 show that the interaction of the treatments led to a more than threefold increase in both the K^+^/Na^+^ and Ca^2+^/Na^+^ ratios, while the rates of increase exceeded ninefold in the Mg^2+^/Na^+^ ratio in the first and second seasons.

**Table 7:** Interactive impact of NFTs and PFTs on K^+^/Na^+^, Ca^2+^/Na^+^ and Mg^2+^/Na^+^ ratios in leaves of sweet potato plants cultivated in saline-calcareous soil during 2022 and 2023 growing seasons.

Treatment		K^+^/Na^+^ Ratio	Ca^2+^/Na^+^ Ratio	Mg^2+^/Na^+^ Ratio
NFTs	PFTs			
2022 growth season				
N_1_	P_1_	45.38±0.02^a^	20.59±0.01^a^	5.47±0.02^a^
	P_2_	10.20±0.02^e^	8.00±0.01^c−e^	0.52±0.04^f^
	P_3_	15.34±0.01^cd^	8.75±0.03^b−d^	2.57±0.02^cd^
N_2_	P_1_	15.21±0.02^cd^	11.0±50.02^b^	2.91±0.02^c^
	P_2_	21.21±0.02^bc^	7.18±0.02^de^	4.60±0.01^b^
	P_3_	11.18±0.03^de^	5.33±0.03^e^	0.78±0.03^ef^
N_3_	P_1_	14.99±0.01^cd^	10.42±0.01^bc^	2.00±0.01^d^
	P_2_	24.39±0.03^b^	9.94±0.02^b−d^	2.58±0.02^cd^
	P_3_	17.22±0.02^cd^	5.71±0.03^e^	1.27±0.02^e^
N * P		**	**	**
2023 growth season				
N_1_	P_1_	47.82±0.01^a^	26.15±0.01^a^	8.61±0.01^a^
	P_2_	10.24±0.04^d^	9.32±0.02^bc^	0.80±0.02^f^
	P_3_	14.56±0.01^b−d^	10.15±0.02^bc^	3.68±0.01^cd^
N_2_	P_1_	14.49±0.01^b−d^	12.68±0.02^b^	4.22±0.02^c^
	P_2_	20.66±0.02^bc^	8.74±0.02^bc^	6.57±0.01^b^
	P_3_	10.49±0.01^cd^	6.22±0.01^c^	1.12±0.02^ef^
N_3_	P_1_	14.13±0.02^b−d^	11.58±0.03^b^	2.85±0.01^c−e^
	P_2_	23.99±0.01^b^	11.88±0.03^b^	3.77±0.01^c^
	P_3_	16.68±0.03^b−d^	6.68±0.01^c^	1.80±0.01^d−f^
N * P		**	**	**

Mean values (±SE), same lower-case letters in each columns denote not significant among treatments according to the Duncan’s Multiple range test at *p*≤0.05. NFT nitrogenous fertilizer type; N_1_, N_2_ and N_3_ represent ammonium nitrate, urea and ammonium sulfate, respectively. PFT phosphorus fertilizer type; P_1_, P_2_ and P_3_ represent granular calcium super phosphate, mono-ammonium-phosphate and urea-phosphate, respectively.

### Physiological and growth characteristics

#### The individual impacts of NFTs

**Fig. 7:**
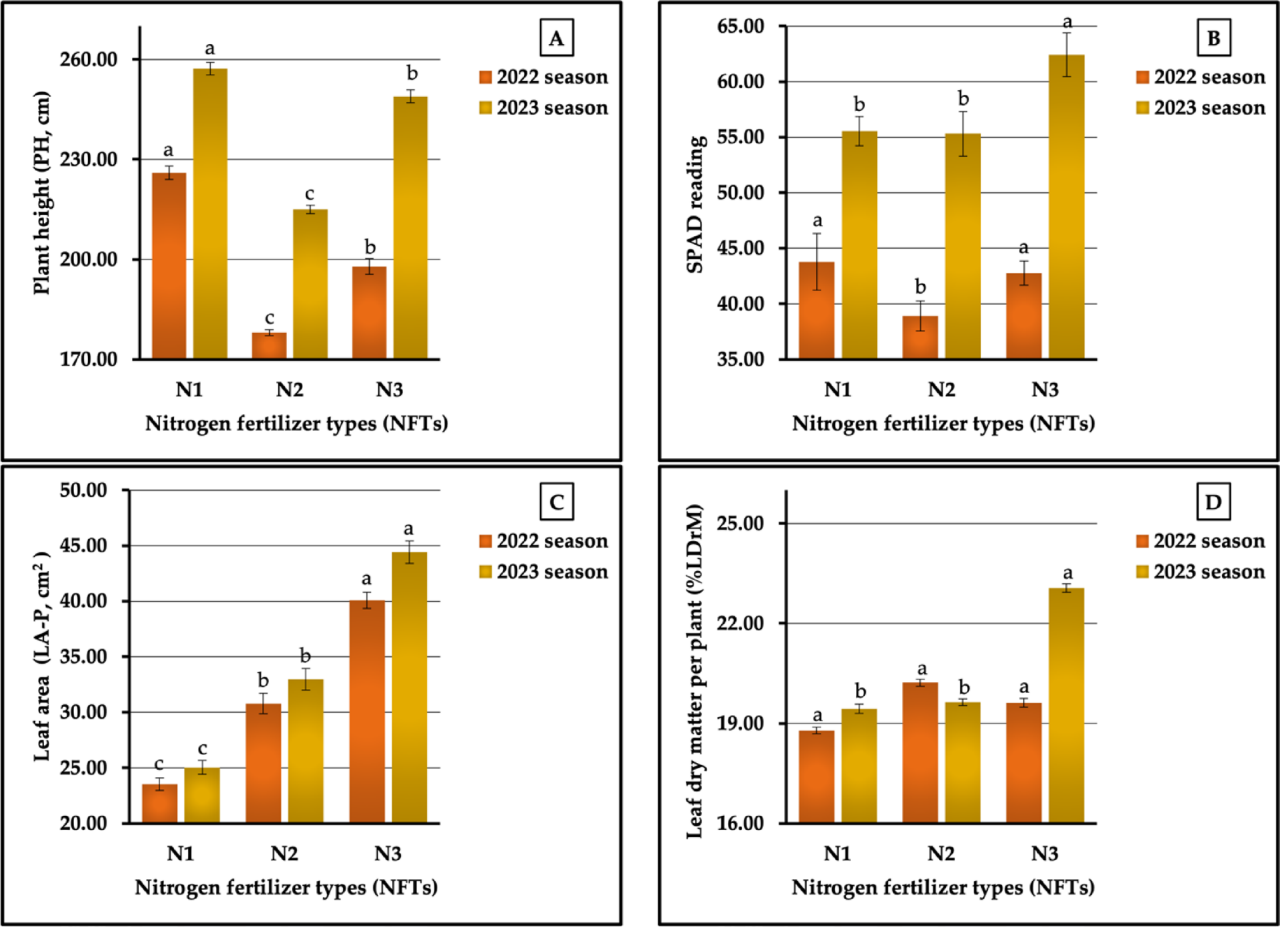
(A-D). The individual impact of nitrogen fertilizer types (NFTs) on **(A)** plant height, **(B)** SPAD reading, **(C)** leaf area, and **(D)** plant dry matter percentage of sweet potato plants cultivated in saline-calcareous soil during 2022 and 2023 growing seasons. Bars sharing the same letter in each columns denote not significantly different among treatments according to the Duncan’s Multiple range test at *p*≤0.05.

The results obtained from our field research, as demonstrated graphically in Figs. 7 (A-D), show that the highest values in plant height (PH-P) and leaf area (LA-P) (226.00 vs. 257.22 cm and 40.08 vs. 44.39 cm^2^ in 2022 and 2023 growing seasons) were measured in the sweet potato plants fertilized with N_1_ and N_3_. Dissimilar data were recorded in both growing seasons regarding some other growth parameters. However, the highest values in SPAD readings, an indicator of relative chlorophyll content (43.78 vs. 62.41), were achieved using ammonium nitrate (N_1_) and ammonium sulfate (N_3_) in both seasons, respectively. Meanwhile, the highest leaf dry matter percentage (%DrM-L) values were recorded in plants treated with urea (N_2_) and N_3_ in both seasons, respectively. On the other hand, the least influential treatment was N_2_, which produced the lowest values in PH-P (131. 22 vs. 215.00 cm) and SPAD readings (38.91 vs. 55.31) in both seasons, respectively, and in %DrM-P in the first growing season (18.79%). N_2_ also produced the lowest values in LA-P (25.53 vs. 25.05 cm^2^ in both seasons and the lowest values in %DrM-P (19.44%) in the second season. As observed from the data obtained, the rates of increase in the highest and lowest values were 26.96 vs.19.64% for PH-P, 12.52 vs. 12.84% for the SPAD reading, and 70.34, vs. 72.21% for LA-P in both growing seasons, respectively. The results obtained from our statistical analysis revealed significant differences (at *p* < 0.01) among the studied nitrogenous fertilizers on PH, LA-P in both seasons, and %DrM-P in the second season, while significant differences (at *p* < 0.05) were found in both growth seasons in SPAD readings, and non-significant differences were found on %DrM-P in the first season.

#### The individual impacts of PFTs

**Fig. 8:**
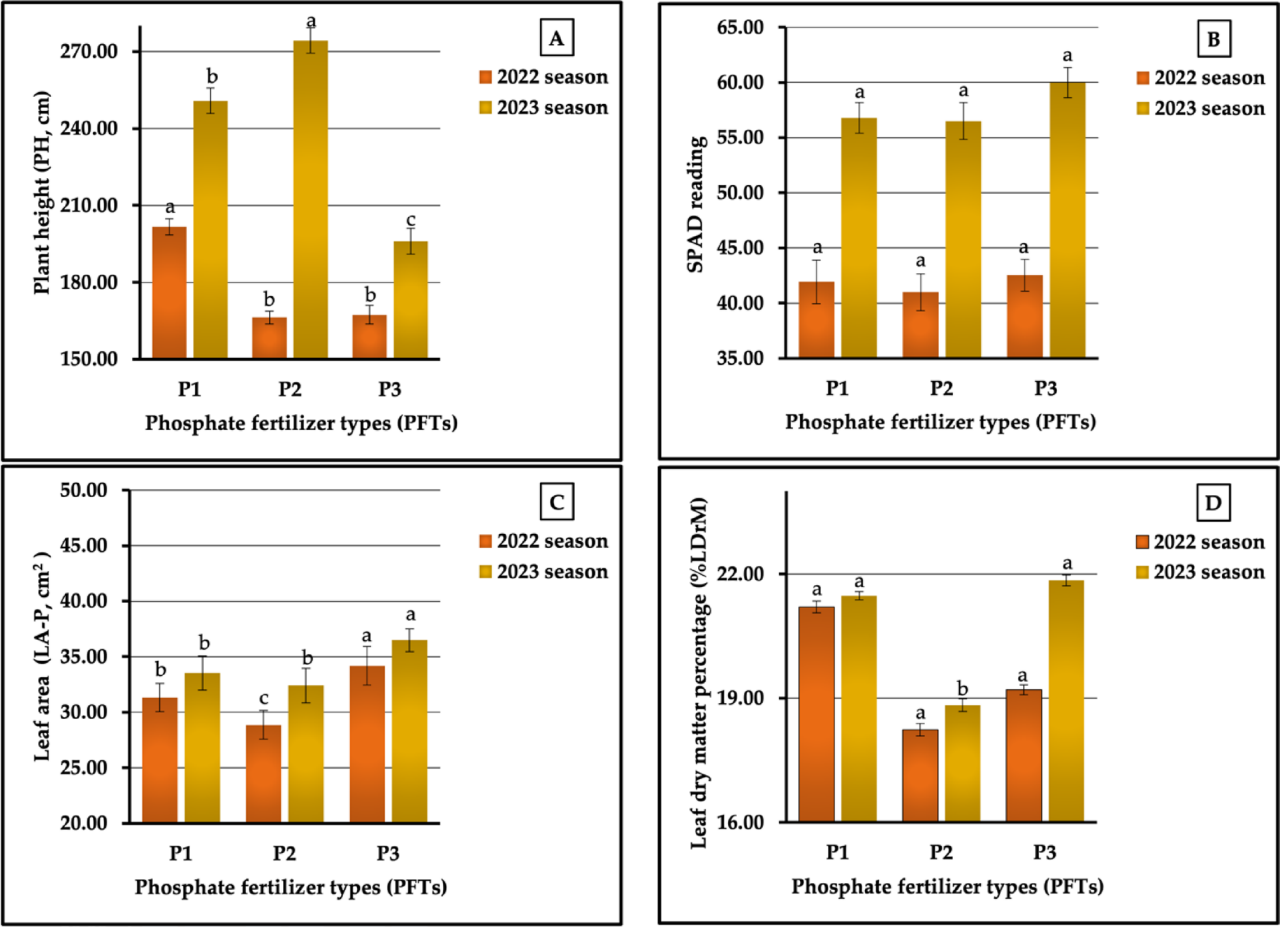
(A-D). The individual impact of phosphate fertilizer types (PFTs) on **(A)** plant height, **(B)** SPAD reading, **(C)** leaf area, and **(D)** plant dry matter percentage of sweet potato plants cultivated in saline-calcareous soil during 2022 and 2023 growing seasons. Bars sharing the same letter in each columns denote not significantly different among treatments according to the Duncan’s Multiple range test at *p*≤0.05.

The results related to the individual impact of the studied phosphorus fertilizers on the aforementioned growth and physiological parameters are graphically represented in Figs. 8 (A-D). The obtained results revealed that treatment with urea-phosphate (P_3_) was the most impactful for the SPAD readings and for LA-P in 2022 and 2023 seasons, during which the highest values were recorded (42.53 vs. 59.98 and 34.18 vs. 36.49 cm^2^, and for %DrM-L in 2023 season (21.85%). In addition, the highest PH-P (201.67 cm) and %DrM-L (21.20%) levels in the first season were recorded in plants treated with N_1_. On the contrary, the lowest values in the SPAD reading (41.00 vs. 56.50), LA-P (28.87 vs. 32.41 cm^2^, and %DrM-L (18.24 vs. 18.33%) in both growth seasons, respectively, and the lowest PH-P (166.22 cm) level in the first season were recorded in plants fertilized with mono-ammonium-phosphate (P_2_). As depicted in Fig. 8 (A-D), the percentages of the increase compared with the lowest values were 21.33 vs. 39.96%, 3.73 vs. 6.16%, 18.39 vs. 12.59%, and 16.23 vs. 16.04% for PH-P, SPAD, LA-P, and %DrM-L in both seasons, respectively. Statistically, the obtained results showed that the treatments had significant differences (at *p* ≤ 0.01) for PH-P and LA-P and non-significant differences for the SPAD readings in both seasons, while the differences in %DrM-P were non-significant and significant in both seasons, respectively.

#### The impact of the NFT X PFT interaction

As demonstrated in Table 8, the analysis of variance showed that highly significant differences were observed for all interactive treatments for all studied physiological and growth parameters, although the results were not consistent for all the aforementioned attributes. The highest PH-P (288.67 vs. 328.00 cm), LA-P (47.97 vs. 52.13 cm^2^, and %DrM-L (28.25 vs. 27.65%) values were obtained in plants treated with ammonium sulfate and mono-ammonium-phosphate (N_3_P_2_), urea-phosphate and calcium superphosphate (N_3_P_1_), and ammonium nitrate and urea phosphate (N_1_P_3_) in both seasons, respectively. Meanwhile, the application of ammonium nitrate with calcium superphosphate (N_1_P_1_) and ammonium sulfate with urea-phosphate (N_3_P_3_) produced the best results in SPAD readings in both seasons, respectively (46.85 vs. 68.02). On the other hand, the lowest PH-P and LA-P values (135.00 vs. 178.00 cm and 18.58 vs. 19.53 cm^2^ in the two growing seasons, respectively, were recorded in plants that received the N_2_P_3_ and N_1_P_1_ treatments. Inconsistent results were obtained for the least influential treatments, since the N_2_P_1_ and N_2_P_2_ treatments produced the lowest values in SPAD readings (36.41 vs. 49.65) and the N_1_P_1_ and N_2_P_3_ treatments produced the lowest values in %DrM-L (13.69 vs. 14.30%) in both seasons, respectively.

**Table 8:** The integrative impact of NFTs and PFTs on some physiological and growth parameters of sweet potato plants cultivated in saline-calcareous soil during 2022 and 2023 growing seasons.

Treatment			PH	SPAD reading	LA-*P*	DrM-*P*
NFT		PFT	(cm)		(cm^2^)	%
2022 growth season						
N_1_	P_1_		228.33±2.03^b^	46.85±2.26^a^	18.58±0.55^g^	13.69±1.82^d^
	P_2_		273.33±0.88^a^	43.07±1.35^a−c^	21.27±0.92^f^	14.43±1.29^d^
	P_3_		176.33±2.33^e^	41.43±1.10^bc^	30.73±0.74^d^	28.25±1.63^a^
N_2_	P_1_		206.67±2.91^c^	36.41±2.05^d^	27.47±0.53^e^	25.25±1.49^ab^
	P_2_		136.67±1.76^f^	37.92±1.38^cd^	26.68±0.48^e^	21.55±1.78^a−c^
	P_3_		190.67±2.40^d^	42.39±1.14^a−c^	38.16±0.76^b^	13.87±1.73^d^
N_3_	P_1_		170.00±2.89^e^	42.53±0.80^a−c^	47.97±0.92^a^	24.65±1.67^ab^
	P_2_		88.67±2.91^g^	42.00±1.12^a−c^	38.65±1.44^b^	18.74±1.20^b−d^
	P_3_		135.00±2.88^f^	43.78±1.28^ab^	33.63±0.74^c^	15.48±1.32^cd^
N * P			**	*	**	**
2023 growth season						
N_1_	P_1_		266.33±0.88^c^	58.67±3.26^b^	19.53±0.46^f^	15.10±1.19^d^
	P_2_		303.67±2.03^b^	52.41±1.88^bc^	22.75±1.13^f^	15.59±1.28^d^
	P_3_		201.67±2.19^f^	55.55±1.10^bc^	32.87±0.82^d^	27.65±1.06^a^
N_2_	P_1_		245.33±1.20^d^	59.92±0.65^b^	28.92±0.77^e^	25.58±1.17^ab^
	P_2_		191.33±2.03^g^	49.65±4.30^c^	28.85±1.05^e^	19.02±1.12^cd^
	P_3_		208.33±1.45^e^	56.37±2.40^bc^	41.15±0.93^c^	14.30±1.90^d^
N_3_	P_1_		240.67±1.86^d^	51.75±2.29^bc^	52.13±1.23^a^	23.72±1.78^a−c^
	P_2_		328.00±1.73^a^	67.45±1.66^a^	45.62±1.99^b^	21.88±1.43^bc^
	P_3_		178.00±2.08^h^	68.02±1.31^a^	35.43±1.39^d^	23.59±1.37^a−c^
N * P			**	**	**	**

Mean values (±SE), same lower-case letters in each columns denote not significant among treatments according to the Duncan’s Multiple range test at *p*≤0.05. NFS nitrogenous fertilizer source; N_1_, N_2_ and N_3_ represent ammonium nitrate, urea and ammonium sulfate, respectively. PFS phosphorus fertilizer source; P_1_, P_2_ and P_3_ represent granular calcium super phosphate, mono-ammonium-phosphate and urea-phosphate, respectively. H-P plant height, SPAD relative chlorophyll content, LA-P leaf area, and %DrM-P plant dry matter percentage.

### Tubers’ nutrient content

#### The individual impact of NFTs

**Fig. 9:**
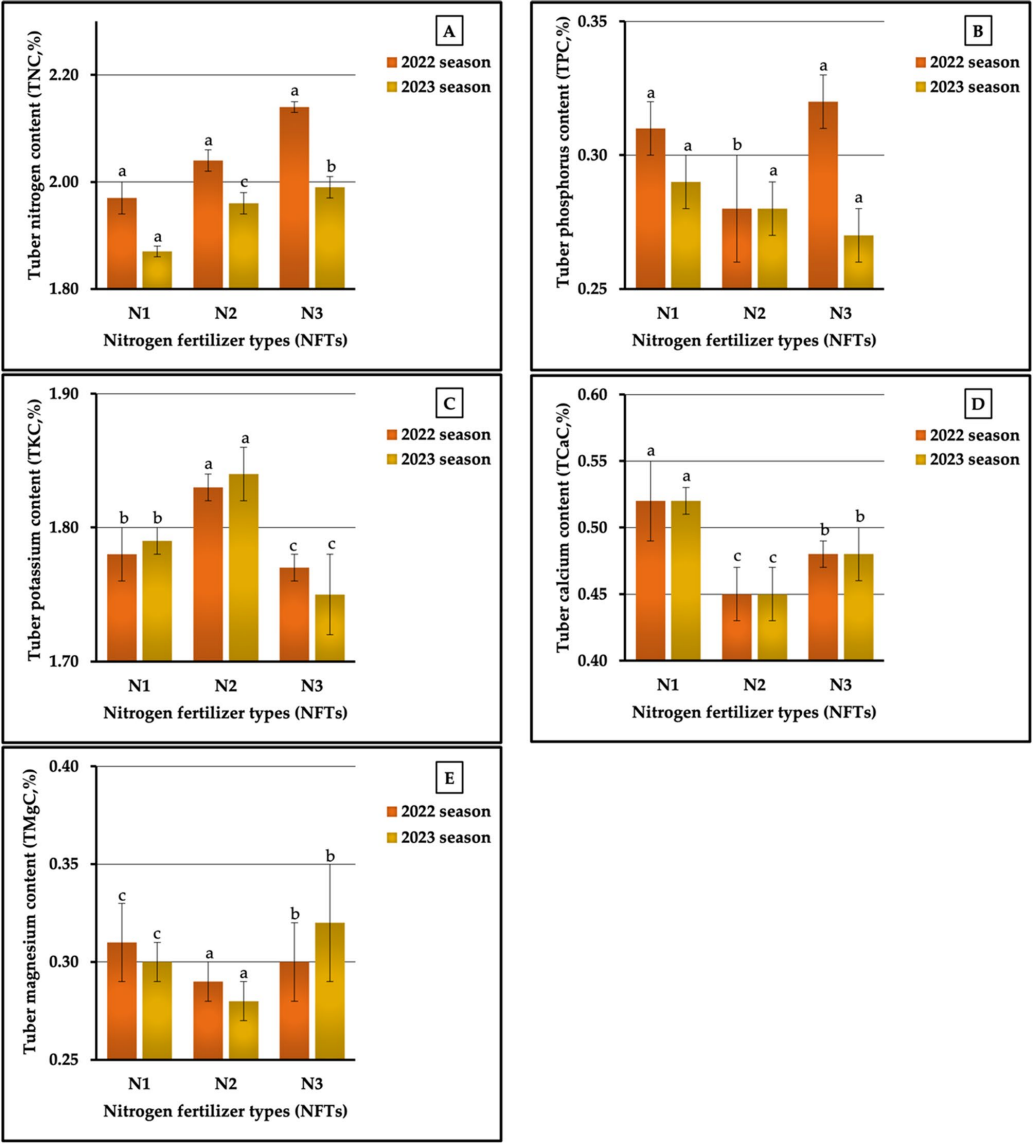
(A-E). The individual impact of nitrogen fertilizer types (NFTs) on tuber macronutrients content; **(A)** nitrogen, **(B)** phosphorus, **(C)** potassium, **D)** calcium, and **E)** magnesium on sweet potato plants cultivated in saline-calcareous soil during 2022 and 2023 growing seasons. Bars sharing the same letter in each columns denote not significantly different among treatments according to the Duncan’s Multiple range test at *p*≤0.05.

The results graphically represented in Fig. 9 (A-E) indicate that N_1_ treatment was the most impactful on tuber calcium (TCaC) and magnesium (TMgC) contents, producing 0.53 vs. 0.52% and 0.31 vs. 0.30% in both seasons. Moreover, N_1_ treatment produced a tuber phosphorus content (TPC) of 0.29% in the second season. Meanwhile, the N_3_ treatment was the most influential on tuber nitrogen content (TNC) in both seasons, recording 2.14 vs. 1.99% in TNC and TPC in the first season, respectively, while the best values in tuber potassium content (TKC) were produced by N₂ (1.83 vs. 1.84% in the two growing seasons, respectively). On the other hand, N₂ was the least impactful on TPC (0.28 vs. 0.27%), TCaC (0.46 vs. 0.45%), and TMgC (0.31 vs. 0.30%) in 2022 and 2023 growing seasons, respectively, while the lowest values in TNC (1.97 vs. 1.87%) and TKC (1.77 vs. 1.75%) were recorded in sweet potato plants treated with N_1_ and N_3_ in both seasons, respectively. Values of 3.63 vs. 6.42%, 14.29 vs. 7.41%, 3.39 vs. 5.14, 15.13 vs. 15.56%, and 14.82 vs. 3.45% were recorded as the increase in percentages for TNC, TPC, TKC, LCaC, and TMgC compared with the lowest values in both seasons, respectively. Statistically, our results indicated that NFSs significantly (at *p* ≤ 0.01) affected TKC, TCaC, and TMgC and had no significant effect on TNC in both seasons, respectively. In addition, NFSs showed significant impacts (at *p* ≤ 0.05) in the first season, and TPC demonstrated no significant effect in the second season.

**Fig. 10:**
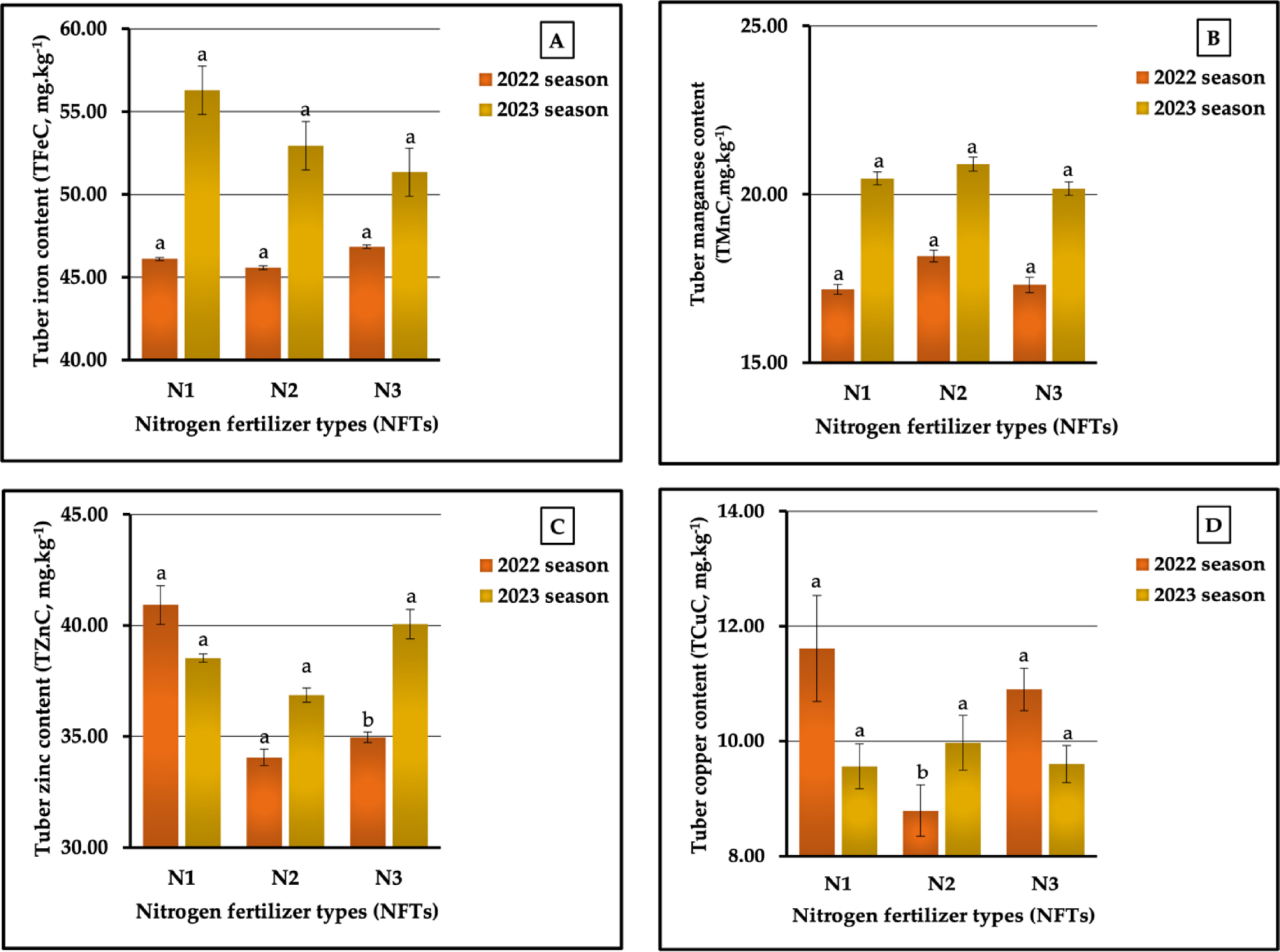
(A-D). The individual impact of nitrogen fertilizer types (NFTs) on tuber micronutrients content; **(A)** iron, **(B)** manganese, **(C)** zinc, and **B)** copper of sweet potato plants cultivated in saline-calcareous soil during 2022 and 2023 growing seasons. Bars sharing the same letter in each columns denote not significantly different among treatments according to the Duncan’s Multiple range test at *p*≤0.05.

Regarding the impact of NFTs on the micronutrient content of tuberous roots, it was noted that the highest tuber manganese content (TMnC) was achieved in plants treated with N₂ (18.17 vs. 20.9.0 mg kg^−1^ in both seasons, respectively), and the highest LCuC was achieved in the second season (9.97 mg kg^−1^).Dissimilar results were found for tuber iron (TFeC), zinc (TZnC), and copper (TCuC) contents, since the N_1_ treatment demonstrated the highest values in TFeC (56.28 mg kg^−1^) in the first season and the highest values in TZnC (40.92 mg kg^−1^) and TCuC (11.61 mg kg^−1^) in the first season. As depicted in Fig. 10 (A-D), the plants fertilized with N_2_ demonstrated the lowest value in TZnC (34.06 vs. 36.87 mg kg^−1^) in both seasons; in addition, these plants demonstrated the lowest values in TFeC (45.58 mg kg^−1^) and TCaC (8.79 mg kg^−1^) in 2022 season. Furthermore, the lowest values in TMnC (17.18 mg kg^−1^) in the first season and in TCuC (9.56 mg kg^−1^) in the first season were recorded in plants that received the N_1_ treatment. Based on a comparison between the highest and lowest values, the rates of increment amounted to 2.76 vs. 9.62% for TFeC, 5.76 vs. 3.62% for TMnC, 20.14 vs. 8.65% for TZnC, and 32.08 vs. 4.29% for TCuC in the first and second seasons, respectively. Statistical analysis showed non-significant effects on both TFeC and TMnC in both seasons and on TZnC and TCuC in the second season. Meanwhile, significant influences (at *p* ≤ 0.05) were found for TZnC and TCuC in the first season.

#### The individual impact of PFTs

**Fig. 11:**
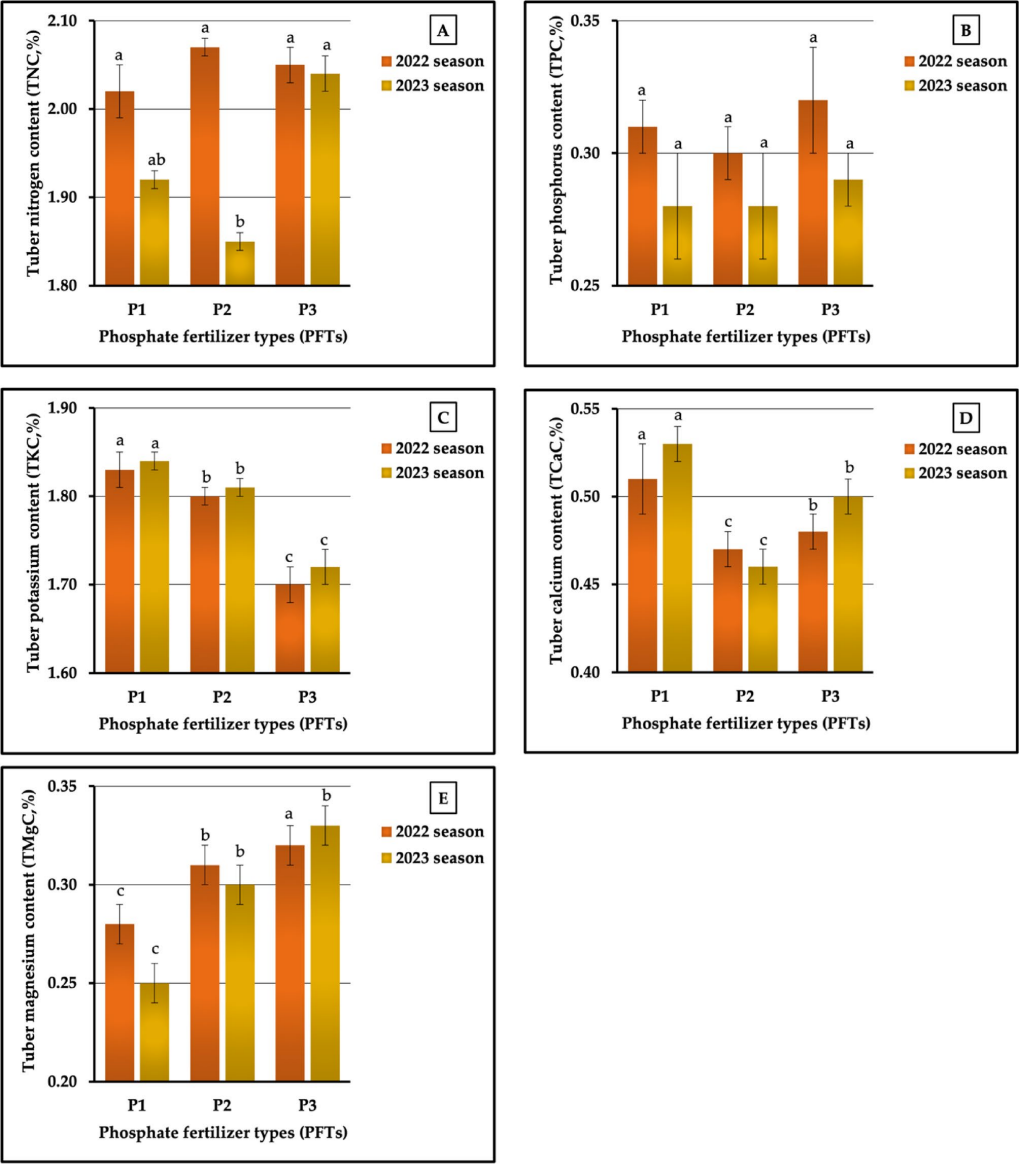
(A-E). The individual impact of phosphate fertilizer types (PFTs) on tuberous root macronutrients content; **(A)** nitrogen, **(B)** phosphorus, **(C)** potassium, **(D)** calcium, and **(E)** magnesium of sweet potato plants cultivated in saline-calcareous soil during 2022 and 2023 growing seasons. Bars sharing the same letter in each columns denote not significantly different among treatments according to the Duncan’s Multiple range test at *p*≤0.05.

The data pertaining to the influence of PFTs are graphically represented in Figs. 11 (A-E). For plants fertilized with P_3_, the highest values recorded were 0.32 vs. 0.29% for TPC and 0.32 vs. 0.33% for TMgC in both seasons, respectively. Similar results were achieved for TKC and TCaC, since the highest values for both of these contents were produced in plants treated with P_1_ in the two growing seasons, respectively. Meanwhile, P₂ and P_3_ produced the best values for TNC (2.07 vs. 2.04% in both seasons, respectively). In contrast, the lowest values recorded in plants treated with P_3_, P₂, and P_1_ in 2022 and 2023 growing seasons, respectively, were 1.70 vs. 1.72% for TKC, 0.47 vs. 0.46% for TCaC, and 0.28 vs. 0.25% for TMgC. Furthermore, the application of P_1_ produced the lowest values in TNC (2.02%) in the first season and the lowest values in TPC (0.28%) in the second season. P₂ treatment was the least impactful for TNC (1.85%) in the second season and for TPC (0.30%) in the first season. The results related to the lowest and highest values indicated that the rates of increase were 2.48 vs. 10.27 for TNC, 6.67 vs. 3.57% for TPC, 7.65 vs. 6.98% for TKC, 8.51 vs. 15.22% for TCaC, and 25.00 vs. 32.00% for TMgC in the first and second seasons, respectively. Statistically, the analysis of variance showed highly significant influences on TKC, TCaC, and TMgC and non-significant impacts on TNC and TPC in both growing seasons, respectively.

**Fig. 12:**
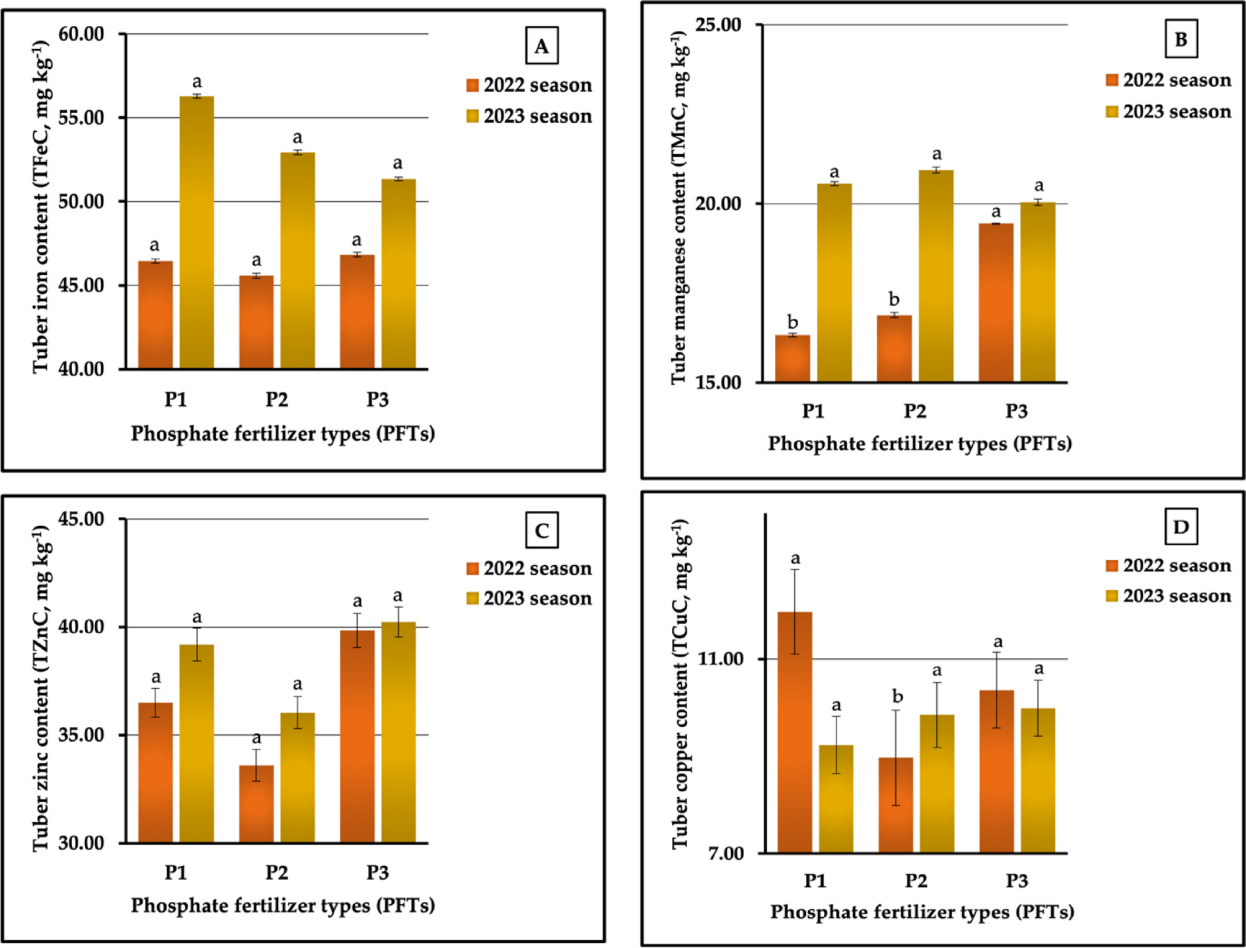
(A-D). The individual impact of phosphate fertilizer types (PFTs) on tuber micronutrients content; **(A)** iron, **(B)** manganese, **(C)** zinc, and **(D)** copper (LCuC) of sweet potato plants cultivated in saline-calcareous soil during 2022 and 2023 growing seasons. Bars sharing the same letter in each columns denote not significantly different among treatments according to the Duncan’s Multiple range test at *p*≤0.05.

The results presented in Fig. 12 (A-D) markedly indicate that the plants fertilized with P_3_ had the greatest values (39.84 vs. 40.23 mg kg^−1^) for TZnC. In addition, these plants had the highest values in TFeC (55.69 mg kg^−1^) and in TCuC (9.99 mg kg^−1^) in the second season, and the highest value in TMnC (19.44 mg kg^−1^) in the first season. Similarly, the P_1_ treatment produced the best values for TFeC (46.45 mg kg^−1^) in the 2022 season and for TCuC (11.97 mg kg^−1^) in 2023 season. On the other hand, P_2_ treatment also produced the lowest values in TFeC in the second season and the lowest in TCuC in the first season. Moreover, the highest levels of TZnC in both seasons were obtained in plants treated with P₂. Similarly, P_3_ produced the lowest values in TFeC (45.98 mg kg^−1^) in the first season and of TMnC (20.04 mg kg^−1^) in the second season. Furthermore, the lowest values in TMnC (16.33 mg kg^−1^) in the first season and the lowest values in TCuC (9.23 mg kg^−1^) in the second season were recorded in plants treated with P_1_. It can be observed from Fig. 12 (A-D) that the percentages of increase amounted to 1.02 vs. 8.71% for TFeC, 19.05 vs. 4.49% for TMnC, 18.57 vs. 11.74% for TZnC, and 33.45 vs. 8.23% for TCuC in the two growing seasons, respectively. The results obtained from the ANOVA showed highly significant impacts on the TMnC and TCuC in 2022 season, no significant effects on TNC and TZnC in both seasons, and no significant impacts on TMnC and TCuC in the second growing season.

#### The impact of the NFT X PFT interaction

Regarding the impact of the interaction between NFS and PFS on tuberous root macronutrient content, our results demonstrated that the application of N₂P_1_, N_1_P_1_, and N₂P_3_ produced the highest values of TKC (2.06 vs. 2.07%), TCaC (0.65 vs. 0.65%), and TMgC (0.33 vs. 0.35%) in 2022 and 2023 growing seasons, respectively, as presented in Table 9. Dissimilar data were obtained for TNC and TPC, as the highest TNC (2.24 vs. 2.11% in both seasons, respectively) was recorded in plants treated with N_3_P_1_ and N_3_P_3_, and the highest value of TPC (0.34 vs. 0.30% in both seasons, respectively) was recorded in plants treated with N_1_P_1_ and N_1_P_3_. As shown in Table 9, the lowest values for TNC (1.84 vs. 1.74%), TPC (0.26 vs. 0.24%), TKC (1.67 vs. 1.69%), TCaC (0.39 vs. 0.43%), and TMgC (0.23 vs. 0.22%) were measured in sweet potato plants fertilized with N_1_P_1_, N₂P_1_, N_2_P_3_, N_1_P_2_, and N₂P_1_ in the first and second seasons, respectively. The different sources of nitrogenous and phosphorus interactions led to significant differences (at *p* ≤ 0.01) for TKC, TCaC, and TMgC and non-significant impacts on TNC in both seasons. Significant influences (at *p* ≤ 0.05) and no significant effects were found for TPC in both seasons, respectively. The increase rates reached 21.74 vs. 21.26%, 30.77 vs. 25.00% 23.35 vs. 22.49%, 66.67 VS. 55.81%, and 43.48 vs. 59.10% for N, P, K, Ca, and Mg contents in 2022 and 2023 growing seasons, respectively.

**Table 9:** The interactive impact of NFTs X PFTs on tuberous root macronutrients content of sweet potato plants cultivated in saline-calcareous soil during 2022 and 2023 growing seasons.

Treatment		Tuber macronutrients content				
NFTs	PFTs	TNC	TPC	TKC	TCaC	TMgC
		(%)				
2022 growth season						
N_1_	P_1_	1.84±0.02^b^	0.34±0.01^a^	1.68±0.02^f^	0.65±0.02^a^	0.29±0.02^d^
	P_2_	2.10±0.01^ab^	0.27±0.01^bc^	1.87±0.03^b^	0.39±0.02^h^	0.33±0.01^a^
	P_3_	1.97±0.03^ab^	0.34±0.02^a^	1.78±0.01^c^	0.52±0.01^c^	0.31±0.02^c^
N_2_	P_1_	1.96±0.03^ab^	0.26±0.02^c^	2.06±0.01^a^	0.41±0.02^g^	0.23±0.03^f^
	P_2_	2.07±0.02^ab^	0.29±0.02^a−c^	1.77±0.01^d^	0.47±0.02^e^	0.32±0.02^b^
	P_3_	2.10±0.02^ab^	0.30±0.02^a−c^	1.67±0.01^g^	0.48±0.03^d^	0.33±0.02^a^
N_3_	P_1_	2.24±0.02^a^	0.32±0.01^ab^	1.77±0.01^d^	0.47±0.02^e^	0.31±0.01^c^
	P_2_	2.04±0.03^ab^	0.34±0.02^a^	1.77±0.02^d^	0.54±0.01^b^	0.28±0.02^e^
	P_3_	2.13±0.03^ab^	0.31±0.01^a−c^	1.71±0.03^e^	0.44±0.02^f^	0.32±0.02^b^
N * P		ns	*	**	**	**
2023 growth season						
N_1_	P_1_	1.74±0.02^b^	0.30±0.02^a^	1.69±0.02^f^	0.66±0.02^a^	0.31±0.02^d^
	P_2_	1.91±0.01^ab^	0.28±0.01^ab^	1.88±0.02^b^	0.37±0.03^h^	0.36±0.02^a^
	P_3_	1.97±0.01^ab^	0.30±0.02^a^	1.79±0.02^c^	0.50±0.01^c^	0.32±0.02^c^
N_2_	P_1_	2.00±0.01^ab^	0.26±0.02^b^	2.07±0.01^a^	0.39±0.7^g^	0.23±0.03^f^
	P_2_	1.81±0.01^ab^	0.27±0.01^ab^	1.76±0.03^d^	0.45±0.02^e^	0.34±0.03^b^
	P_3_	2.06±0.02^ab^	0.28±0.02^ab^	1.68±0.03^g^	0.49±0.02^d^	0.35±0.02^a^
N_3_	P_1_	2.03±0.03^ab^	0.28±0.01^ab^	1.76±0.02^d^	0.46±0.01^e^	0.32±0.01^c^
	P_2_	1.83±0.01^ab^	0.30±0.02^a^	1.78±0.02^d^	0.53±0.02^b^	0.29±0.02^e^
	P_3_	2.11±0.01^a^	0.29±0.02^ab^	1.70±0.01^e^	0.42±0.01^f^	0.33±0.02^b^
N * P		ns	ns	**	ns	**

Mean values (±SE), different lower-case letters in each columns denote significant among treatments according to the Duncan’s Multiple range test at *p*≤0.05. NFS nitrogenous fertilizer source; N_1_, N_2_ and N_3_ represent ammonium nitrate, urea and ammonium sulfate, respectively. PFS phosphorus fertilizer source; P_1_, P_2_ and P_3_ represent granular calcium super phosphate, mono-ammonium-phosphate and urea-phosphate, respectively. TNC, TPC, TKC, TCaC, and TMgC indicate the tuberous root nitrogen, phosphorus, potassium, calcium, and magnesium content, respectively.

As shown in Table 10, there was no consistent trend regarding the influence of NFT_S_ and PFT_S_ interaction on tuber micronutrient content. However, the highest values of TFeC (47.58 vs. 62.96 mg kg^−1^), TMnC (22.25 vs. 21.41 mg kg^−1^), TZnC (47.63 vs. 47.90 mg kg^−1^), and TCuC (13.14 vs. 10.70 mg kg^−1^) were produced in plants treated with N_3_P_3_ and N_1_P_3_, N₂P_3_ and N₂P₂, N_1_P_1_ and N_3_P_3_, and N_1_P_3_ and N_3_P₂ in both seasons, respectively. On the contrast, the N₂P₂ produced the lowest values (30.34 vs. 34.10 mg kg^−1^) in TZnC in both seasons and the lowest value in TFeC (44.63 mg kg^−1^) in the first season. Moreover, plants treated with N_3_P_3_ produced the lowest values in TFeC (48.41 mg kg^−1^) and TMnC (18.96 mg kg^−1^) in the second season. Meanwhile, N_2_P_3_ and N_3_P_1_ treatments were the least influential on TCuC, which was recorded as 6.86 vs. 8.53 mg kg^−1^ in both seasons, respectively. The results obtained from the statistical analysis showed non-significant differences for all aforementioned nutrients except TMnC and TCuC in the first season. All the previous treatments produced increment rates in both seasons of 6.6 vs. 30.06%, 48.53 vs. 21.92%, 56.99 vs. 25.81, and 91.58 vs. 25.44% for TFeC, TMnC, TZnC, and TCuC in the first and second seasons, respectively.

**Table 10:** The interactive impact of NFTs X PFTs on tuberous root micronutrients content of sweet potato plants grown in saline-calcareous soil during 2022 and 2023 growing seasons.

Treatment		Tuber micronutrients content			
NFTs	PFTs	TFeC	TMnC	TZnC	TCuC
		(mg kg^−1^)			
2022 growth season					
N_1_	P_1_	46.84±0.01^a^	17.16±0.01^bc^	47.63±0.01^a^	11.75±0.03^ab^
	P_2_	46.34±0.01^a^	15.33±0.02^cd^	34.96±0.02^ab^	9.93±0.02^a−d^
	P_3_	45.15±0.01^a^	19.04±0.02^b^	40.18±0.01^ab^	13.14±0.01^a^
N_2_	P_1_	46.91±0.02^a^	14.94±0.01^d^	30.38±0.02^b^	11.24±0.03^a−c^
	P_2_	44.63±0.01^a^	17.31±0.02^bc^	30.34±0.01^b^	8.27±0.02^cd^
	P_3_	45.20±0.01^a^	22.25±0.01^a^	41.45±0.01^ab^	6.86±0.02^d^
N_3_	P_1_	45.61±0.01^a^	16.87±0.01^b−d^	31.49±0.02^b^	12.92±0.02^a^
	P_2_	47.32±0.01^a^	18.04±0.01^b−d^	35.51±0.02^ab^	8.70±0.03^b−d^
	P_3_	47.58±0.02^a^	17.02±0.02^b−d^	37.89±0.01^ab^	11.08±0.02^a−c^
N * P		ns	ns	*	*
2023 growth season					
N_1_	P_1_	56.69±0.02^ab^	20.26±0.01^a^	41.56±0.02^a^	9.34±0.02^a^
	P_2_	49.17±0.01^b^	20.17±0.02^a^	38.54±0.02^a^	9.18±0.01^a^
	P_3_	62.96±0.01^a^	20.98±0.01^a^	39.82±0.01^a^	10.15±0.01^a^
N_2_	P_1_	52.63±0.02^ab^	21.10±0.01^a^	38.54±0.01^a^	9.83±0.02^a^
	P_2_	50.44±0.02^ab^	21.41±0.02^a^	24.10±0.02^a^	9.69±0.02^a^
	P_3_	55.71±0.03^ab^	20.17±0.01^a^	37.98±0.02^a^	10.38±0.01^a^
N_3_	P_1_	51.55±0.02^ab^	20.33±0.02^a^	37.48±0.01^a^	8.53±0.01^a^
	P_2_	54.07±0.02^ab^	21.24±0.01^a^	39.80±0.03^a^	10.70±0.02^a^
	P_3_	48.41±0.01^b^	18.96±0.01^a^	42.90±0.02^a^	9.58±0.02^a^
N * P		ns	ns	ns	ns

Mean values (±SE), same lower-case letters in each columns denote not significant among treatments according to the Duncan’s Multiple range test at *p*≤0.05. NFS nitrogenous fertilizer source; N_1_, N_2_ and N_3_ represent ammonium nitrate, urea and ammonium sulfate, respectively. PFS phosphorus fertilizer source; P_1_, P_2_ and P_3_ represent granular calcium super phosphate, mono-ammonium-phosphate and urea-phosphate, respectively. TFeC, TMnC, TZnC, and LCuC indicate the tuberous root iron, manganese, zinc, and copper content, respectively.

### Tubers’ physical measurements

#### The individual impact of NFTs

**Fig. 13:**
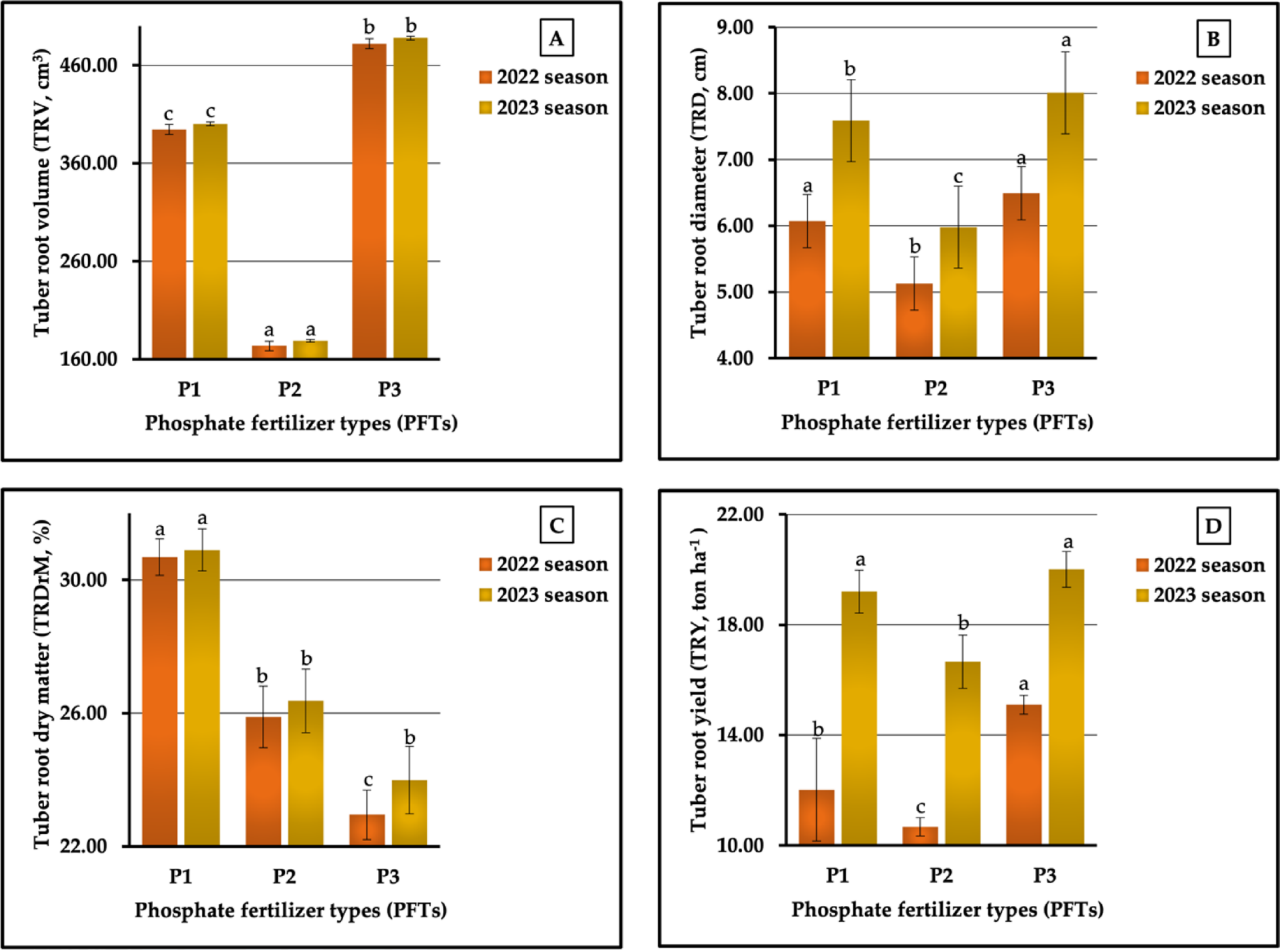
(A-D). The individual impact of phosphate fertilizer types (PFTs) on **(A)** Tuber root volume, **(B)** tuber root diameter, **(C)** tuber dry matter percentage, and **(D)** tuber root yield of sweet potato plants cultivated in saline-calcareous soil during 2022 and 2023 growing seasons. Bars sharing the same letter in each columns denote not significantly different among treatments according to the Duncan’s Multiple range test at *p*≤0.05.

The results, as observed in Fig. 13 (A-D), showed that the highest tuberous root volume (TRV) values were obtained in sweet potato plants treated with N_2_ in 2022 and 2023 seasons (401.67 vs. 407.78 cm³). For plants receiving this treatment, the highest values in tuberous root diameter (TRD) were recorded in the second season (7.46 cm). Similar results were obtained for tuberous root dry matter percentage (%DrM-T), as plants fertilized with N_1_ had the highest values (30.68 vs. 30.10%) in both seasons, respectively. Moreover, its profound effect was clearly demonstrated by the fact that the highest TRD value was recorded in the first season (5.97 cm). Dissimilar results were obtained for tuberous root yield (TRY); however, the highest values were achieved in plants treated with N_3_ and N_1_ in both growing seasons, respectively (14.51 vs. 20.09 ton ha^−1^). On the other hand, our results demonstrated that the plants fertilized with N_1_ produced the lowest values (281.78 vs. 287.11 cm³) in TRV in both seasons, respectively, and the lowest values in TRD (6.93 cm) in the second season. Meanwhile, the lowest value (5.78 cm) in TRD in the second season and the lowest value in %DrM-T in both seasons (23.06 vs. 24.75%) were recorded in the plants fertilized with N_3_. This treatment was the least influential on TRY (10.68 vs. 16.45 t ha^−1^ in the first and second seasons, respectively). The results that we obtained, shown in Fig. 13 (A-D), indicate that the rates of increment compared with the lowest values were 42.55 vs. 42.03% for TRV, 3.29 vs. 7.65% for TRD, and 35.86 vs. 22.13% for TRY in both growing seasons, respectively. The analysis of variance indicated that all NFTs had a significant influence (at *p* ≤ 0.01) on TRV and significant effects (at *p* ≤ 0.05) on %DrM-T, non-significant and significant differences on TRD, and highly significant and significant differences in TRY in both growing seasons, respectively.

#### The individual impact of PFTs

**Fig. 14:**
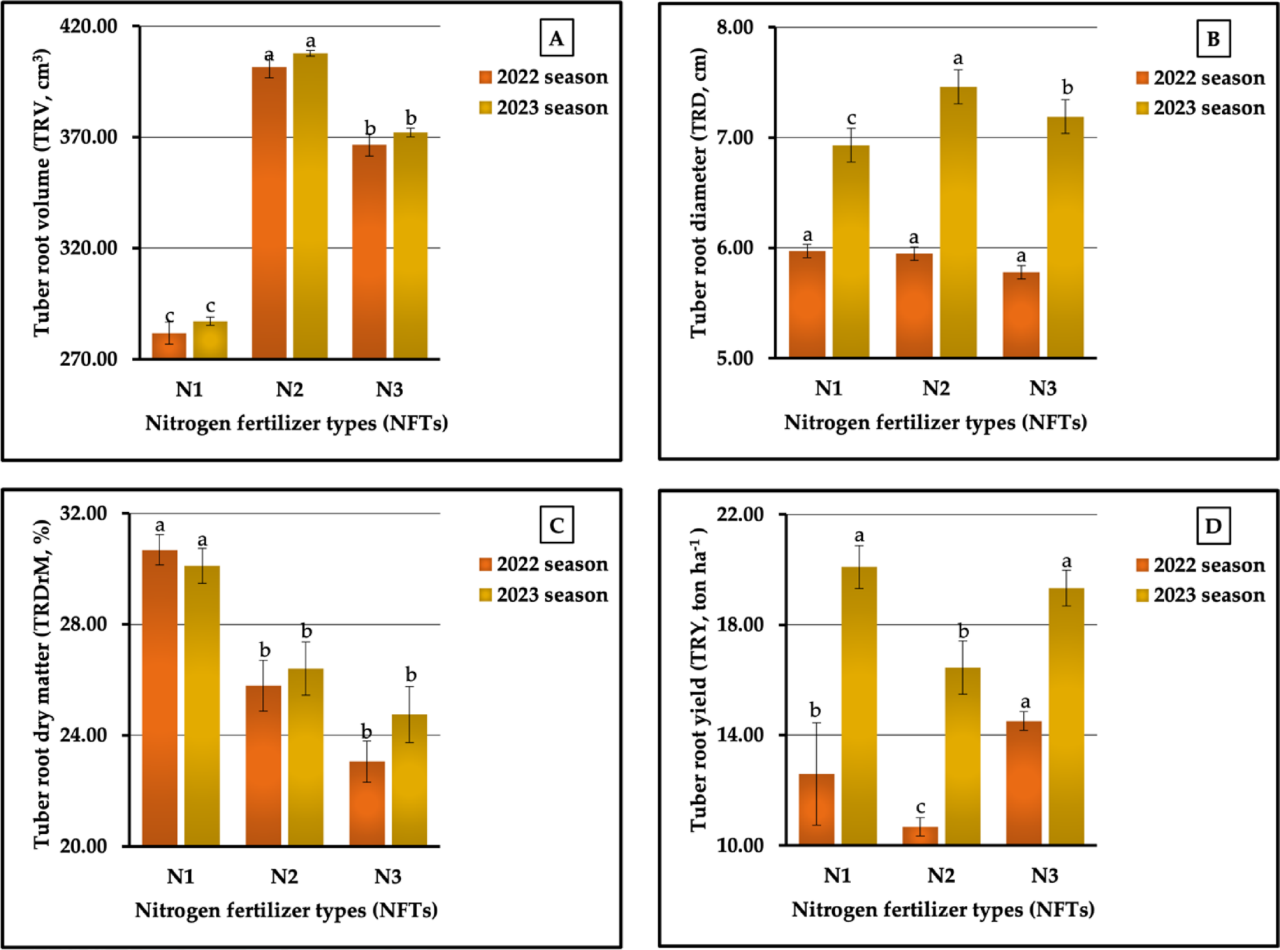
(A-D). The individual impact of nitrogen fertilizer types (NFTs) on **(A)** tuber root volume, **(B)** tuber root diameter, **(C)** tuber root dry matter, and **(D)** tuber root yield of sweet potato plants grown in saline-calcareous soil during 2022 and 2023 growing seasons. Bars sharing the same letter in each columns denote not significantly different among treatments according to the Duncan’s Multiple range test at *p*≤0.05.

The results graphically represented in Fig. 14 (A-D) indicate that P_3_ had a clear effect on TRV, TRD, and TRY in the first and second seasons. The results revealed that TRV, TRD, and TRY, in descending order, were ranked as follows: P_3_ ˃ P_1_ ˃ P₂, recording 482.00 cm^3^ > 394.44 cm^3^ > 173.56 cm^3^ and 487.78 cm^3^ > 400.22 cm^3^ > 179.00 cm^3^ for TRV, 6.49 cm > 6.07 cm > 5.13 cm and 8.01 cm > 7.59 cm > 5.98 cm for TRD, and 15.10 t ha^−1^ > 12.02 t ha^−1^> 10.67 t ha^−1^ and 20.01 t ha^−1^ > 19.20 t ha^−1^ > 16.66 t ha^−1^ for TRY in both seasons, respectively. Meanwhile, for %DrM-T, the PFTs were ranked in descending order as P_1_ (30.69 vs. 30.90%) > P₂ (25.89 vs. 26.37) > P_3_ (22.95 vs. 23.99) in the two growing seasons, respectively. Based on the highest and lowest values, the rates of increase reached 177.71 vs. 172.50% for TRV, 25.66 vs. 33.95% for TRD, 33.73 vs. 28.80% for % DrM-T, and 41.92 vs. 20.11 for TRY in 2022 and 2023 seasons, respectively. The analysis of variance showed a highly significant effect on all studied parameters in both growth seasons except %DrM-T in the second season. Significantly different impacts between the various PFT treatments were found.

#### The interactive impact of the NFTs and PFTs

According to the results presented in Table 11, the plants treated with N_3_P_3_ produced the best values in TRV (669.00 vs. 675.00 cm³) and TRD (7.20 vs. 8.94 cm) in both growth seasons and in TRY in the first season (18.61 ton ha^−1^). In the second season, the highest TRY value was achieved in plants treated with N_1_P_3_. Furthermore, N_1_P_1_ was the most impactful treatment on the %DrM-T in both seasons, respectively, recording 36.86 vs. 34.35%. Dissimilar data were produced for the lowest values; however, plants fertilized with N_3_P_2_ produced the lowest values in TRV (127.00 vs. 132.33 cm³) and in %DrM-T (21.34 vs. 22.21%) in both growth seasons and the lowest values in TRD (5.54 cm) in the second season. Meanwhile, plants nourished with N₂P₂ demonstrated the lowest values in TRY (10.12 vs. 15.58 ton ha^−1^) in the two growing seasons and the lowest values in TRD (4.57 cm) in the first season. It can be observed from Table 11 that the increasing rates of the highest and lowest values were 426.77 vs. 410.10% for TRV, 57.55 vs. 61.37% for TRD, 72.73 vs. 54.66 for %DrM-P, and 83.89 vs. 52.76% for TRY in the first and second seasons, respectively. Statistically, highly significant variations were found among the studied characters in both seasons except for in %DrM-T in the second season.

**Table 11:** The interactive impact of NFTs X PFTs on some tubers’ physical measurement and tuber root yield sweet potato plants cultivated in saline-calcareous soil during 2022 and 2023 growing seasons.

Treatment		TRV	TRD	DrM-T	TRY
NFTs	PFTs	(cm^3^)	(mm)	(%)	(ton ha^−1^)
2022 growth season					
N_1_	P_1_	319.67 ± 0.03^d^	6.12 ± 0.2^b−d^	36.86 ± 0.03^a^	10.55 ± 0.04^de^
	P_2_	258.67 ± 0.02^g^	6.03 ± 0.03^b−d^	32.90 ± 0.03^a^	11.35 ± 0.04^d^
	P_3_	267.00 ± 0.02^f^	5.75 ± 0.02^cd^	22.26 ± 0.02^b^	15.59 ± 0.04^b^
N_2_	P_1_	560.00 ± 0.02^b^	6.75 ± 0.02^ab^	32.38 ± 0.02^a^	11.12 ± 0.04^d^
	P_2_	135.00 ± 0.03^h^	4.57 ± 0.03^e^	23.41 ± 0.02^b^	10.12 ± 0.05^e^
	P_3_	510.00 ± 0.04^c^	6.52 ± 0.03^a−c^	21.59 ± 0.01^b^	10.81 ± 0.05^de^
N_3_	P_1_	303.67 ± 0.03^e^	5.35 ± 0.02^de^	22.82 ± 0.01^b^	14.38 ± 0.04^c^
	P_2_	127.00 ± 0.03^i^	4.80 ± 0.03^e^	21.34 ± 0.02^b^	10.55 ± 0.04^de^
	P_3_	669.00 ± 0.02^a^	7.20 ± 0.03^a^	25.01 ± 0.02^b^	18.61 ± 0.04^a^
N * P		**	**	**	**
2023 growth season					
N_1_	P_1_	325.33 ± 0.03^d^	7.20 ± 0.03^d^	34.35 ± 0.04^a^	18.33 ± 0.02^cd^
	P_2_	264.00 ± 0.03^g^	6.77 ± 0.04^g^	33.71 ± 0.03^ab^	18.14 ± 0.02^cd^
	P_3_	272.00 ± 0.04^f^	6.83 ± 0.04^f^	22.23 ± 0.04^c^	23.80 ± 0.01^a^
N_2_	P_1_	566.33 ± 0.03^b^	8.49 ± 0.04^b^	32.90 ± 0.03^ab^	17.13 ± 0.03^de^
	P_2_	140.67 ± 0.02^h^	5.65 ± 0.04^h^	23.20 ± 0.03^c^	15.58 ± 0.01^e^
	P_3_	516.33 ± 0.03^c^	8.26 ± 0.03^c^	23.13 ± 0.04^c^	16.64 ± 0.02^de^
N_3_	P_1_	309.00 ± 0.02^e^	7.09 ± 0.03^e^	25.43 ± 0.02^c^	22.15 ± 0.01^b^
	P_2_	132.33 ± 0.03^i^	5.54 ± 0.04^i^	22.21 ± 0.02^c^	16.24 ± 0.01^e^
	P_3_	675.00 ± 0.03^a^	8.94 ± 0.03^a^	26.61 ± 0.03^bc^	19.59 ± 0.01^c^
N * P		**	**	*	**

Mean values (±SE), same lower-case letters in each columns denote not significant among treatments according to the Duncan’s Multiple range test at *p*≤0.05. NFTs nitrogenous fertilizer types; N_1_, N_2_ and N_3_ represent ammonium nitrate, urea and ammonium sulfate, respectively. PFTs phosphorus fertilizer types; P_1_, P_2_ and P_3_ represent granular calcium super phosphate, mono-ammonium-phosphate and urea-phosphate, respectively. TRV, and TRD indicate the tuber root volume, and diameter. DrM-T and TRY indicate the tuber root dry matter percentage and tuber root yield, respectively.

### Pearson’s correlation and Stepwise multiple regression analysis

Meanwhile, a significant (*p* ≤ 0.05) positive correlation (*r* = 0.458*, 0.393*, 0.401*, and 0.068*) was observed between TRY and LNaC, LFeC, LMnC, and TRY, respectively, in the first growing season and (*r* = 0.426*) between TRY and LCaC in the second season (Table 12). On the other hand, TRY correlated negatively (*r* = −0.123, −0.519, and − 0.256) with LMgC, PH-P, and %DrM-T, respectively, in the first season and correlated negatively (*r* = −0.057, −0.061, −0.026, −0.090, −0.132, −0.250, −0.100, and − 0.088) with LKC, LMgC, LFeC, LMnC, LCuC, PH-P, the SPAD readings, and %DRM-T, respectively, in the second season. With regard to ion homeostasis, we found that TRY correlated negatively with K^+^/Na^+^ (*r* = −0.247 and − 0.135) and Mg^2+^/Na^+^ (*r* = −0.338 and − 0.159) in both growing seasons, respectively.

**Table 12:** Pearson’s correlation coefficient between tuberous root yield (TRY) with 13 selected parameters of sweet potato plants fertilized with nitrogen and phosphorus fertilizer types under saline-calcareous soil during 2022 and 2023 seasons.

Parameter	2022 growth season		2023 growth season	
	Pearson *r*	Probabilities	Pearson *r*	Probabilities
Leaf nutrients content				
LNC	0.327	0.096	0.583**	0.001
LPC	0.538**	0.004	0.220	0.270
LKC	0.364	0.062	−0.057	0.777
LCaC	0.167	0.406	0.426*	0.027
LMgC	−0.123	0.540	−0.061	0.761
LNaC	0.458*	0.016	0.203	0.309
LFeC	0.393*	0.042	−0.026	0.897
LMnC	0.401*	0.038	−0.090	0.654
LZnC	0.056	0.782	0.161	0.422
LCuC	0.171	0.394	−0.132	0.512
Growth-physiological attributes				
PH-P	−0.519	0.006	−0.250	0.209
SPAD reading	0.170	0.397	−0.100	0.619
LA-P	0.311	0.115	0.203	0.311
%DrM-L	0.183	0.362	0.495**	0.009
Ion’s homeostasis				
K^+^/Na^+^	−0.247	0.215	−0.135	0.501
Ca^2+^/Na^+^	−0.279	0.159	0.003	0.988
Mg^2+^/Na^+^	−0.358	0.067	−0.159	0.427
Quality characters				
TRV	0.068*	0.014	0.073	0.716
TRD	0.363	0.063	0.168	0.403
%DrM-T	−0.256	0.197	−0.088	0.662

LNC, LPC, LKC, LCaC, LMgC, LNaC, LFeC, LMnC, LZnC, and LCuC indicate the leaf nitrogen, phosphorus, potassium, calcium, magnesium, sodium, iron, manganese, zinc and copper contents, respectively. PH-P, SPAD reading, LA-P, and %DrM-L indicate the plant height, relative chlorophyll content, leaf area, and leaf dry matter percentage, respectively. TRV, TRD, and %DrM-T indicate the tuberous root volume, diameter and dry matter percentage, respectively.

The data presented in Table 13 refer to a stepwise regression analysis to determine the relationship between TRY and the controlled related traits of sweet potato plants grown in saline calcareous soil. The results that we obtained indicate that the adjusted *R*^2^ values were 0.604 (*r* = 0.806) for model 3. This revealed that 60.4% of the variations in TRY occurred due to variations in the combination of LPC, LCaC, and LCuC in the first season, while in the second season, the adjusted *R*^2^ was 0.859 (*r* = 0.938) for model 4, which revealed that about 85.9% of variations in TRY were obtained because of variations in the combination of LNC, PH-P, LCaC, and LNaC.

**Table 13:** Proportional contribution in predicting tuberous root yield (TRY) using Stepwise multiple linear regression for sweet potato plants fertilizes with nitrogen and phosphate fertilizer types under saline-calcareous conditions during 2022 and 2023 growing season.

Model	Entered variable	Intercept	b_1_	b_2_	b_3_		*r*	*R* ^2^	Adj. *R*^2^	SEE
2022 growth season										
1	LPC	6.404^**^	3.651^**^				0.538^a^	0.289	0.261	2.49
2	LCaC	−2.141^ns^	5.005^**^	3.376^**^			0.690^b^	0.476	0.432	2.18
3	LCuC	−17.604^**^	5.563^**^	5.346^**^	0.329^**^		0.806^c^	0.650	0.604	1.82
2023 growth season										
			b_1_	b_2_	b_3_	b_4_				
1	LNC	6.326^**^	3.372^**^				0.583^a^	0.340	0.314	2.29
2	PH-P	9.399^**^	4.471^**^	−0.029^**^			0.764^b^	0.583	0.549	1.86
3	LCaC	3.199^**^	4.576^**^	−0.034^**^	3.403^**^		0.911^c^	0.830	0.808	1.21
4	LNaC	−1.356^**^	4.874^**^	−0.029^**^	3.292^**^	11.499^**^	0.938^d^	0.881	0.859	1.04

*=*p* ≤ 0.05 and **=*p* ≤ 0.01. LPC leaf phosphorus content, LCaC leaf calcium content LCuC leaf copper content, LNC leaf nitrogen content, LNaC leaf sodium content, and PH plant height. r correlation coefficient, R^2^ coefficient of determination, Adj. R^2^ adjusted R2, SEE standard error of estimates.

Our results showed that model 3 in the first season, and model 4 in the second season, were the most suitable fit due to their high adjusted *R*^2^ values. However, these models’ adjusted *R*^2^ (0.604 and 0.859) values had a higher adjusted R^2^ and a lower standard error (SEE): 1.82 and 1.04 in the first and second seasons, respectively. In the first season, TRY = −17.604 + 5.563 LCuC + 5.346 LCaC + 0.329 LPC in the first season and TRY = −1.356 + 4.874 LNaC − 0.029 LCaC + 3.292 PH-*P* + 11.499 LNC in the second season.

## Discussion

### The individual impact of NFTs

In arid and semi-arid regions, most arable lands are prone to one or more abiotic stresses; these stresses may reduce the productivity of most crops by as much as 70%^45^. Among abiotic stresses, salinity and calcification are the most severe environmental factors that can restrict plant growth and productivity^46^. Improving the nutritional status of plants under these brutal conditions is one of the strategies developed by plant nutrition scientists. As presented in Figs. 1-A and 2-C, the highest values in leaf nitrogen content (LNC) in both seasons and leaf zinc content (LZnC) in the first season were achieved in plants fertilized with ammonium nitrate (NH_4_NO_3_); however, the appropriate ratio of NH_4_^+^: NO_3_^−^ improved root development, including root length, root tips, surface volume, and area of roots, which, in turn, stimulated N uptake^47^. The same authors reported that applying N fertilizer that included an appropriate amount of NH_4_ had beneficial effects on root architecture, including increased root surface area and root length, that promote the uptake of some mineral nutrients. Due to plant nitrogen acquisition, taller plants were observed Fig. 7-A, as N plays a significant role in the formation of cells and tissues and in improving vegetative growth through cell division and elongation^48^. Relevant studies have pointed out that mixing NH_4_^+^ and NO_3_^−^ was more influential for plant growth than a single form of nitrogen^49,50^. Our results indicated the significant superiority of ammonium sulfate (N_3_) in improving the absorption of P, K, Ca, and Cu in the first and second seasons and in improving leaf manganese content (LMnC) in the first season and leaf zinc content (LZnC) in the second season. In a previous study^51^, reported the profound impact of NH_4_^+^-N in reducing the pH of rhizosphere soil, which, in turn, increased the availability of P.

In general, these improvements in nutrient uptake in sweet potato plants treated with N_3_ might be due to the profound effect of (NH_4_)_2_SO_4_ in lowering the pH in the vicinity of roots^52,53^. However, plant roots release protons (H^+^) when NH_4_^+^ ions are absorbed to maintain a constant intracellular charge balance, resulting in a decrease in rhizospheric pH^54^. Furthermore, NH_4_^+^-containing fertilizer has a chemical acidifying influence in the rhizosphere zone resulting from the nitrification process, since protons are released in this process^55^. This, in turn, could improve the solubility, availability, and absorption of micronutrients. In other words, NO_3_^−^ had the opposite impact of NH_4_^+^. However, when plants uptake H^+^ by co-transportation across the plasma membrane of root cells, leading to an increase in rhizospheric pH due to OH- being released when NO_3_^−^ is absorbed, a corresponding decrease in nutrient uptake is observed^56^. However, our results regarding the LKC in sweet potato plants were not matched by those of^57^, who documented that the uptake of K decreased in high concentrations of NH_4_^+^. This is likely because the NH_4_^+^ was absorbed, inducing acidification of the rhizosphere soil, which resulted in an increase in K content. Another possible explanation was reported by^53^, who reported that at the lower soil pH, H^+^ ions are considered a strong competitor with exchangeable K^+^, therefore replacing it in the soil solution. Moreover, we found a close relationship between LCaC and LKC, depending on the improving impact of Ca^2+^ on the uptake of K^+^. As expected, in our research, the application of urea did not act as a good source for nitrogen under undesirable conditions (pH of 7.54 vs. 7.65, EC of 7.74 vs. 8.90, and CaCO_3_ of 19.50 vs. 19.20%). However, its addition increased the LMgC in sweet potato plants. Despite the fact that urea is the richest fertilizer in N, containing up to 46%, it is the least effective fertilizer in comparison with other nitrogenous fertilizers, as urea is hydrolyzed by urease enzymes as soon as it is added to soil. These enzymes can be directly converted to NH_4_^+^ before they can be taken up by plants^58,59^. In our study, Mg content was high due to the competitive uptake between Mg^2+^ and Ca^2+^ (antagonism effect)^60^. Very recently^61^, documented that urea is the least acceptable among primary N fertilizers because N is lost in many pathways through fixation, volatilization, denitrification, and leaching. However, ammonia (NH_3_) is converted into ammonium (NH_4_^+^) and nitrate (NO_3_^−^). These results were in good accordance with recent, previously reported studies by^62^, who documented that the N losses are the greatest due to the rapid conversion of NH_2_-N to NH_4_-N in the soil solution by urease enzymes. This, in turn, elevates pH in the rhizosphere zone and elevates micronutrient availability.

In our present research, the results indicate that the application of NH_4_NO_3_ is beneficial to the leaf area (LA-P) in both seasons and beneficial to the SPAD readings and %DrM-L in the second season due to the positive effect of the appropriate NH_4_:NO_3_ratio. Our obtained results were in line with those in previous studies; however^63–65^, admitted that the appropriate NH_4_^+^:NO_3_^−^ ratio plays a significant role in photosynthesis, in absorption of nutrients and water, and in biomass of several plant species, while the improvements resulting from (NH_4_)_2_SO_4_ were closely associated with lower leaf sodium content (LNaC). Improved plant growth was observed through SPAD readings, LA-P, and %DrM-L attributes (Fig. 9A-D). These attributes greatly contributed to better tuberous root yield. An increase in the SPAD reading can promote the photosynthetic rate of sweet potato plants, to which is attributed an enhancement in the plants’ efficient use of light energy in the transformation of CO_2_ and mineral N into plant materials, which eventually increases the TRY. These results were in agreement with previous studies by^66^, who documented that the application of N significantly increased SPAD readings and photosynthetic rate. This, in turn, markedly enhanced physiological growth attributes, which ultimately increased the yield.

### The individual impact of PFTs

As previously documented, P is also an important nutrient in agricultural production systems^67^. In our research, we found that plants fertilized with urea-phosphate produced the highest values in all studied macronutrients and micronutrients except leaf Ca, Mg, and Cu contents in both seasons. In addition, the application of mono-ammonium-phosphate (MAP) was the superior treatment for LCuC in the first and second seasons. These findings are likely due to the enhanced effect of both UP and MAP in lowering rhizosphere pH. Meanwhile, the UP had a more profound pH-lowering impact than MAP, as the average pH values of UP and MAP were 1.8 and 4.5, as presented in Table 4. In this context, this appreciable decrease in soil pH was the main factor influencing the increase in solubility and availability of nutrients^68^. Consistent with the findings of our research^69,70^, also documented the significant role of phosphoric acid (H_3_PO_4_) in lowering rhizosphere pH. Another likely reason for this is the pronounced effect of UP and MAP in increasing the root hairs, which would enhance the uptake efficiency of sweet potato plants. The decreases in LCaC and LMgC could be attributed to HPO_4_^−−^ and H_2_PO_4_^−^ ions being fixed as a result of high soil pH (7.54 vs. 7.65) and CaCO_3_ (19.50 vs. 19.20%), as shown in Table 1. Nevertheless, applying UP followed by MAP produced the best results. The highest LCaC and LMgC values were obtained in plants treated with calcium superphosphate (CSP). One possible reason for this was that the HPO_4_^2−^ could quickly react with Ca^2+^ ions in the soil to form insoluble calcium phosphate according to the following equation: Ca(H_2_PO_4_) + 2 Ca^2+^ → Ca_3_ (PO_4_)_2_ + 4 H^+^. As our results may further explain, this likely occurs because the H_2_PO_4_^−^ and HPO_4_^−−^ ions produced by UP and MAP are more soluble and are taken up easily by plant roots compared with CSP. In other words, better results were achieved in plants fertilized with either UP or MAP (compared to CSP) due to presence of N and P in one chemical structure, as shown in Table 4. Furthermore, the observed decrease in LCuC might be due to the antagonistic effect between Cu and other micronutrients such as Fe, Mn, and Zn^68^.

As graphically represented in Fig. 8(A-D), significant enhancements in physiological growth attributes and in SPAD readings were observed in sweet potato plants fertilized with UP (in terms of leaf area) in both seasons; the same was observed for % DrM-L in the second season. In addition, the highest values in plant height (PH-P) were obtained in plants treated with MAP in the second growing seasons. Furthermore, no significant improvements in % DrM-L were observed in the first season. The interpretation of these results depends on the improvement observed in the absorption of nutrients. However, adequate soil nutrients can enhance the competitiveness of nutrients in roots^71,72^, resulting in an increase in the number of roots, which, in turn, improves nutrient uptake. Another possible explanation for plant growth is the presence of amide nitrogen (NH_2_-N) in the chemical structure of UP: plants convert this into NH_4_^+^ and then NO_3_^−^. In addition, the presence of ammonical nitrogen (NH_4_-N) in MAP is converted by plants into NO_3_: this has considerable contributions in increasing root length, root surface area, and root volume. All these improvements ameliorated the efficiency of the absorption of nutrients by roots. The findings of^73^ Zhu et al.`s study in 2021 demonstrated that NO_3_^−^ions lowered the pH in the rhizosphere zone. In line with previous studies^74^, documented that applying an appropriate amount of PF, irrespective of its type, markedly enhanced the activity of N-metabolism-related enzymes and improved N metabolism.

Notably, UP-treated plants produced the highest values in TRV, TRD, and TRY. This is clear evidence that TRY is closely associated with nutrient content and physiological growth traits. These results could be attributed to the cooperative role of N with P in improving plant growth and promoting chlorophyll content, which, in turn, increases flowering^75^. This is similar to what was found in^76^ Kumar et al.‘s work in 2015. In general, improvements in the chlorophyll content of plants subjected to soil that became saline calcareous due to the application of P fertilizers mitigated the stressful effect resulting from high salinity. However, P was more effective for the uptake of particular nutrients, such as Mg, that integrated elements of chlorophyll’s molecular structure^77^. In their previous study^78^, indicated that enhancements in PH-P, LA-P, and %DrM-L were closely associated with the pivotal role of P in increasing cell division and elongation. Moreover, P enhanced the N, K, and Zn uptake. Another potential reason was postulated by^68^, who reported that UP may mobilize the native P in soils through a chelating reaction and that phosphate ions in UP’s chemical structure exerted a positive effect on the availability of some micro elements such as Fe, Mn, and Zn. The close relationship between nutritional status and physiological growth attributes, on the one hand, and tuberous root yield and its components was observed in our present work, since the highest values of TRV, TRD, and TRY were markedly achieved in plants fertilized with UP. Numerous studies have shown that the observed enhancement in TRV and TRD occurred due to the significant role of P in promoting growth and nitrogenase activity^79,80^. These results were in line with the findings of^81^, who documented that the application of P improved the yield-related growth attributes of soybean plants. In other words, these enhancements could be attributed to the synergistic effect between P and the translocation and availability of other nutrients, as adding UP and MAP increased LKC and LZnC^82^. This is reflected in the TRY levels in relevant trials. These results were in agreement with the findings of^83^, who documented that N, P, and K act as cofactors to promote total carbohydrates and their assimilation, which, in turn, increased TRY and attributes related to its quality.

### The impact of the NFT X PFT interaction

The combined impact of N and P effectively contributed to enhancing nutrient uptake and ensuring boosted productivity. In this study, we report promising findings as a result of this combination. Our results indicate that the combined application of NFTs with PFTs contributes considerably to enhancing leaf nutrients content. However, plants treated with urea-phosphate (P_3_) with N, irrespective of its source, produced the highest values of N, P, K, and Zn in both seasons, as well as in LMnC in the first season. These results may be attributed to the fact that the application of P_3_ effectively contributed to reducing the rhizosphere pH^84^. In addition, these results may be attributed to the paramount role of P in enhancing root growth, which increases the uptake of macro- and micronutrients and has a significant effect in enhancing soil microbial activities^67^. Our findings were consistent with the previous results of Salih et al. 2015, who documented that the appropriate application of P fertilizer markedly enhanced leaf nutrient content. Meanwhile, the joint application of N₂ as a N fertilizer source with P, irrespective of its source, significantly increased LMgC, LFeC, and LCuC in both seasons and LPC and LZnc in the second season. These results might occur because the absorption of NH_4_^+^ by sweet potato plants reduced the rhizosphere pH. Moreover, the uptake of NH_4_^+^ caused apoplast acidification due to the activation of plasma membrane H + ATPase mediated by an AMT-dependent NH_4_^+^ mechanism^85^.

Improved plant growth, including improved PH-P, LA-P, and % DrM.L, was observed as a result of applying N_3_P_2_, N_3_P_1_, and N_1_P_3_ in both seasons, respectively. N fertilizer, irrespective of its type, plays a crucial role in enhancing the growth of photosynthetic organs at the vegetative growth stage and increases plant biomass^86^. Additionally, the application of P can directly contribute to enhancing the biomass of aboveground vegetation and physiological characteristics^87^. Interestingly, similar results are produced by the application of P_1_ combined with NFTs, irrespective of their type, as P_1_ consists of phosphorus and calcium in its chemical structure, exerting, therefore, a stabilizing effect on pH. These results were in agreement with previous studies by^88^, who reported that the application of P to soybean plants enhanced root length and dry weight^89^suggested that NH_4_^+^ uptake seemed to be a key factor in the decrease in the apoplast and thus reduces the rhizosphere pH. Enhanced SPAD chlorophyll readings led to an increase in photosynthetic rate, which greatly contributed to increasing the efficient use of light energy in transformation and inorganic N, ultimately increasing productivity.

These results were very consistent with those obtained by other authors^90,91^, who noted that both nitrogen and phosphorus together play a vital role in plant growth and development, including in seedling height, root growth, and biomass accumulation. In this study, we found that improvements related to growth and physiological attributes could be attributed to the combined application of N and P, as this combination was most effective compared with the individual effect of each of them alone. These findings were also in accordance with past studies which reported that the combination of N and P resulted in a significant enhancement in the biomass of Korean pine seedlings^92,93^.

## Conclusion

The general trend demonstrated in our results is that soils with undesirable characteristics can be cultivated by applying an appropriate fertilization strategy that aims to improve nutrient absorption. Crop productivity can be maximized by promoting nutritional status, which, in turn, improves growth and morphological–physiological characteristics and, ultimately, has positive effects on yield. In conclusion, in both seasons, our findings indicated that the application of either mono-ammonium phosphate (P_2_) or urea phosphate (P_3_) (as a phosphate source) with ammonium nitrate (N_1_) or ammonium sulfate (N_3_) (as a nitrogen source) is preferable due to their acidic effects, which are considered more suitable for Egyptian soils compared with calcium super-phosphate fertilizer (P_1_), which is fixed in relatively large quantities under Egyptian soil conditions. However, under Egyptian soil conditions with high alkalinity, we do not recommend using urea fertilizer, which is considered one of the most harmful environmental pollutants due to its residual effects and role in increasing pH, which reduces the absorption of nutrients.

## Data Availability

The datasets used and analyzed during the current study available from the corresponding author on reasonable request.
